# Body weight distortions in an auditory-driven body illusion in subclinical and clinical eating disorders

**DOI:** 10.1038/s41598-022-24452-7

**Published:** 2022-11-21

**Authors:** Ana Tajadura-Jiménez, Laura Crucianelli, Rebecca Zheng, Chloe Cheng, Judith Ley-Flores, Mercedes Borda-Más, Nadia Bianchi-Berthouze, Aikaterini Fotopoulou

**Affiliations:** 1 DEI Interactive Systems Group, Department of Computer Science and Engineering, Universidad Carlos III de, Av. de La Universidad, 30, 28911 Madrid, Leganés, Spain; 2 UCL Interaction Centre (UCLIC), University College London, London, UK; 3 Department of Neuroscience, Karolinska Institutet, Stockholm, Sweden; 4 Department of Clinical, Educational and Health Psychology, University College London, London, UK; 5 Departamento de Personalidad, Evaluación y Tratamiento Psicológico, Universidad de Sevilla, Seville, Spain

**Keywords:** Perception, Human behaviour

## Abstract

Previous studies suggest a stronger influence of visual signals on body image in individuals with eating disorders (EDs) than healthy controls; however, the influence of other exteroceptive sensory signals remains unclear. Here we used an illusion relying on auditory (exteroceptive) signals to manipulate body size/weight perceptions and investigated whether the mechanisms integrating sensory signals into body image are altered in subclinical and clinical EDs. Participants’ footstep sounds were altered to seem produced by lighter or heavier bodies. Across two experiments, we tested healthy women assigned to three groups based on self-reported Symptomatology of EDs (SED), and women with Anorexia Nervosa (AN), and used self-report, body-visualization, and behavioural (gait) measures. As with visual bodily illusions, we predicted stronger influence of auditory signals, leading to an enhanced body-weight illusion, in people with High-SED and AN. Unexpectedly, High-SED and AN participants displayed a gait typical of heavier bodies and a widest/heaviest visualized body in the ‘light’ footsteps condition. In contrast, Low-SED participants showed these patterns in the ‘heavy’ footsteps condition. Self-reports did not show group differences. The results of this pilot study suggest disturbances in the sensory integration mechanisms, rather than purely visually-driven body distortions, in subclinical/clinical EDs, opening opportunities for the development of novel diagnostic/therapeutic tools.

## Introduction

The last two decades have seen a significant increase in research on body dissatisfaction and body image concerns. The concept of body image includes at least two components: one is related to the accuracy in perceiving one’s own body size and shape (perceptual body image), whereas the other component targets one’s own feelings and thoughts towards the shape and size of the body (attitudinal body image)^1,2^ (see review^3^). Despite this increased interest, the co-occurrence of perceptual (e.g., body weight/size overestimations) and attitudinal body image distortions remains debated, mainly because it is unclear whether these two facets of body image are independent or strongly linked to each other^2,4^.

Disturbances in the way in which one's body weight or shape is experienced and evaluated, is one of the diagnostic criteria for Anorexia Nervosa (AN) (5th ed.; DSM-5^5^), an Eating Disorder (ED) characterised by restricting eating and an obsessive fear of gaining weight. Previous studies have shown that people with AN as well as people with body image concerns often perceive their body visually distorted in terms of shape and size^3^, and that they also present negative attitudes towards their bodies^6–8^ (see reviews^9,10^). Distortions in the perceptual body image are not limited to visual body perception and have also been found in the tactile perception^11^ and affordance perception^12^. Furthermore, the intensity and persistence of both attitudinal and perceptual body image distortions are considered negative prognostic factors for long-term outcome in both AN^13^, and they persist even after otherwise successful treatment (i.e., clinical recovery and weight-restoration for at least 1 year^14–16^. The contribution of attitudinal and perceptual body image distortions to AN is so crucial that it has recently been suggested that AN should be re-defined as a disorder of body image^17^. Nevertheless, whether experiencing body image distortions and concerns may represent a risk factor for the development of AN is still debated.

It has been observed that it is common for young girls and adolescents to experience ED symptoms, including weight and shape concerns as well as perceptual and attitudinal body image distortions, without developing an ED^18,19^. However, these body image concerns and related distortions in body image often go undiagnosed in the general population^20^, despite having an important impact on social, motor and emotional functioning^20–24^. This evidence points to the critical need for more research not only on body image distortions in EDs, but also on people experiencing EDs symptomatology and body image concerns, beyond any clinical diagnosis^25,26^. Currently, the mechanisms underlying the onset of such body image distortions and, more generally, the way in which the body is mentally represented in people with ED symptomatology remain unknown.

Recent neuroscientific developments showed that mental body-representations, which include both representations of body image^27^ and representations of body parts position and kinematics (known as “body schema”^28^), are not fixed but are rather dynamic and that they continuously update through the integration of multisensory bodily cues^29,30^. Proof of this is that they can indeed be experimentally changed via multisensory integration paradigms. For instance, observing from a first-person perspective 3D images of different body types, one may get the illusion of owning slim or obese bodies^31^. Another experimental paradigm widely used in research is the Rubber Hand Illusion (RHI)^32^. In the RHI, participants acquire ownership of a rubber hand that is stroked in synchrony with their own hand, which is out of view. Thus, the RHI results from the interplay between visual, tactile, and proprioceptive bodily cues^33^ (see review^34^). In this regard, Eshkevari and colleagues^35^ showed that during the RHI women with AN had a stronger experience of body ownership towards the fake hand compared to healthy controls, and that such experience was significantly related to ED psychopathology. Moreover, another study showed a reduction of the overestimations of own hand width in people with AN after RHI induction^36^. In both studies, the AN group also showed an enhanced experience of ownership over the fake hand in the control asynchronous condition, which usually should not give rise to the RHI. These studies suggest that bodily information is differently processed by people with AN than by controls. The unexpected finding related to the induced RHI in the asynchronous condition suggests that people with AN might have a heightened sensitivity to visual capture^35–37^ (see similar results in neurological patients by Martinaud et al.^38^); that is, seeing a rubber hand from the first person perspective in a congruent position is sufficient for the illusion to occur. However, it remains unclear whether such body-representation malleability in AN is driven by an overreliance on visual cues or rather by a disturbance in the mechanisms integrating exteroceptive sensory signals into body image.

Indeed, our perception of body size or weight may be influenced by other non-visual sensory cues, for instance, the sound of our own footsteps. The heavier the body is, the more energy contained in the low frequency components of the produced footstep sounds^39^. It appears that when we walk, we seemingly integrate these auditory feedback with other sensory signals from the body to form multisensory representations of our body size/weight^40^. Nevertheless, it is only in the last few years that this auditory and multisensory nature of body image has attracted scientific interest. Recent studies have shown that auditory feedback can also update body-representations^40–47^. For instance, altering in real-time the frequency components of the footsteps sounds produced by people as they walk, to make these sounds consistent with those produced by a lighter or heavier body, can result in changes in how people mentally represent their own body size and weight^40,48–52^. However, to our knowledge, this “footstep illusion” has never been tested in clinical and subclinical populations characterized by different levels of self-reported Symptomatology of EDs (SED).

Here, we used the “footsteps illusion” to manipulate body size/weight perceptions in order to measure changes in perceptual and attitudinal body image in relation to different levels of SED (Experiment 1) and in women with AN (Experiment 2). We measured perceptual body image distortions by means of visual and behavioural outcome measures, and also employed questionnaires targeting currently present feelings and attitudes towards one’s own perceived body. The aim of this pilot study was to establish whether individuals with heightened subclinical ED symptomatology (Experiment 1) and individuals with AN (Experiment 2) show an overreliance on auditory bodily information, by showing more distinct changes in perceptual and attitudinal body image in response to altered auditory feedback as compared to healthy controls (i.e., people with lower and medium levels of ED symptomatology). An enhanced auditory bodily illusion in these populations as compared to control groups would suggest an overreliance on exteroceptive bodily signals in general, while changes in body images incongruent with the signalled information would rather suggest a disturbance in the mechanisms integrating exteroceptive sensory signals into body image.

### Approach and hypotheses

Experiments 1 and 2 tested the effects of altered sound bodily feedback (i.e., “High Frequency” and “Low Frequency” footstep sounds) in the body-representation of healthy individuals with various degrees of symptomatology of ED (Experiment 1) and individuals with AN (Experiment 2). Note that three groups of participants were defined in Experiment 1 (Low, Medium and High Symptomatology of ED) as, being this the first study testing the effects of altered sound bodily feedback in body-representation according to ED symptomatology, we aimed to target participants with various degrees of ED symptomatology. Based on previous studies^40,49,52^, we predicted that augmenting the “High frequency” components of the walking sounds will make people represent their body as thinner and lighter, while augmenting the “Low frequency” components of the walking sounds will make people represent their body as wider and heavier. These changes may be accompanied by behavioural and attitudinal/emotional changes, i.e., when perceiving one’s body as lighter, one may feel more positive about this perceived body and walk as if they were lighter, by accelerating movements of the lower limbs and gait speed^53,54^. By contrast, perceiving one’s body as heavier may result in a longer heel strike, slower and less accelerated movements^53^. These behavioural changes in turn may also affect emotional arousal and dominance, i.e., when accelerating one’s movements one may feel aroused^54–56^. In keeping with previous studies investigating bodily illusions in AN (see Introduction), we predicted that such effects would be stronger in individuals with heightened ED symptomatology (which we refer to as High-SED) and AN in comparison to individuals with low and medium ED symptomatology (which we refer to, respectively, as Low-SED and Medium-SED). That is, just like in the case of visual input, people with AN or High-SED will show an over-reliance on auditory signals, thus showing an enhanced experience of the footstep illusion as compared to people with Low- and Medium-SED. The difference in effects would be more pronounced between the High- and Low-SED participants than between the Medium- and Low-SED participants. Alternatively, if our target population relies on auditory bodily cues to update body image just as other population groups do, that is by integrating exteroceptive auditory signals together with other available bodily signals^29,30^, then we will observe similar changes in perceptual and attitudinal body image in individuals with High-SED and AN, as well as in individuals with Low- and Medium-SED. A third alternative is that our target population will not rely at all on (or even reject) the auditory bodily cues, thus we expect our sound manipulations to lead to changes in the experience of the body in ways unrelated to the auditory signal (e.g., bigger represented body with “High Frequency” sound) but perhaps in line with their own beliefs of body size and shape. This third alternative would signal a disturbance in the mechanisms integrating sensory signals into body image. Given that the present work describes a pilot study, we would like to stress the explorative nature of the present experiment as well as the need to describe multiple working hypotheses.

To quantify the effects of auditory feedback on body image, we used three outcome measures: (1) participants’ visually represented body weight and size (*body-visualization tool*), (2) participants’ body movements and gait patterns consistent with having a lighter or heavier body (*gait biomechanics)* and (3) participants’ body feelings and emotional states (*self-report questionnaires*). Importantly, although three outcome measures are expected to show similar patterns of changes in our task, the third measure could show independent effects as it targets explicit, attitudinal components of body-representation (i.e., feelings towards one’s own perceived body), while the other two measures target perceptual components of body-representation (i.e., visual and behavioural measures that quantify the effect of the auditory manipulation on body-representation via non-auditory channels, namely the visual body perception and the movement pattern, respectively), and these two facets are known to dissociate in clinical conditions such as EDs^1,57–59^ as well as in healthy participants^60^.

## Material and methods

### Experiment 1: Effects of altered auditory feedback in healthy individuals with low, medium and high symptomatology of ED

#### Participants

Fifty-eight healthy participants (Mean age ± s.d.: 21.8 ± 3.2 years; age range: 18–32 years), naïve to the study aim, were recruited through the University College London (UCL) research participation system. Similarly to previous studies^35,37,61,62^, only female participants were tested due to the higher prevalence of subclinical and clinical disordered eating and body image concerns in women^31^, as well as the fact that such concerns in men may regard different aspects of the body, e.g., muscularity rather than weight. Participants were pre-screened for disordered eating and body image concerns by means of the Eating Disorders Examination Questionnaire (EDE-Q 6.0^63,64^). Participants that, according to their EDE-Q global score, fell into any of the groups we had defined for the study (Low-, Medium or High-SED, see online supplementary material for inclusion, pre-screening and group assignment criteria) were invited to take part in the study. We recruited three groups of participants with Low-SED (N = 21; EDE-Q score: Mean = 0.27, SD = 0.11, range: 0.14–0.44), Medium-SED (N = 19; EDE-Q score: Mean = 1.5, SD = 0.36, range: 0.9–2.15) and High-SED (N = 18; EDE-Q score: Mean = 3.76, SD = 0.59, range: 2.99–5.19). There were no significant differences in age between groups.

In addition to EDE-Q scores, body weight and height data were measured during the testing day, which allowed calculating the participant’s body-mass index (BMI). The Mean body weight and height (SD) of participants were 58.22(10.92) Kg and 164.19(5.72) cm. The BMI (SD) of the participants in the three SED groups were: Low-SED: 19.75(2.81); Medium-SED: 21.14(1.59); High-SED: 24.09(4.41). There were significant differences between groups in BMI (*F*(2,57) = 9.65, *p* < 0.001). In particular, the High-SED group was significantly different from the Low-SED group (*t*(37) = 3.72, *p* = 0.001) and from the Medium-SED group (*t*(35) = 2.74, *p* = 0.01), while the difference between Low- and Medium-SED groups did not reach significance (*t*(38) = 1.89, *p* = 0.066). Importantly, all the participants had a BMI within the healthy range (18.5–24.9).

All participants gave their informed consent prior to their inclusion in the studies and received compensation for travel expenses and time. The experiment was conducted in accordance with the ethical standards laid down in the 1964 Declaration of Helsinki, as revised in 2008, and approved by the UCL ethics committee.

#### Apparatus and materials

**Figure 1:**
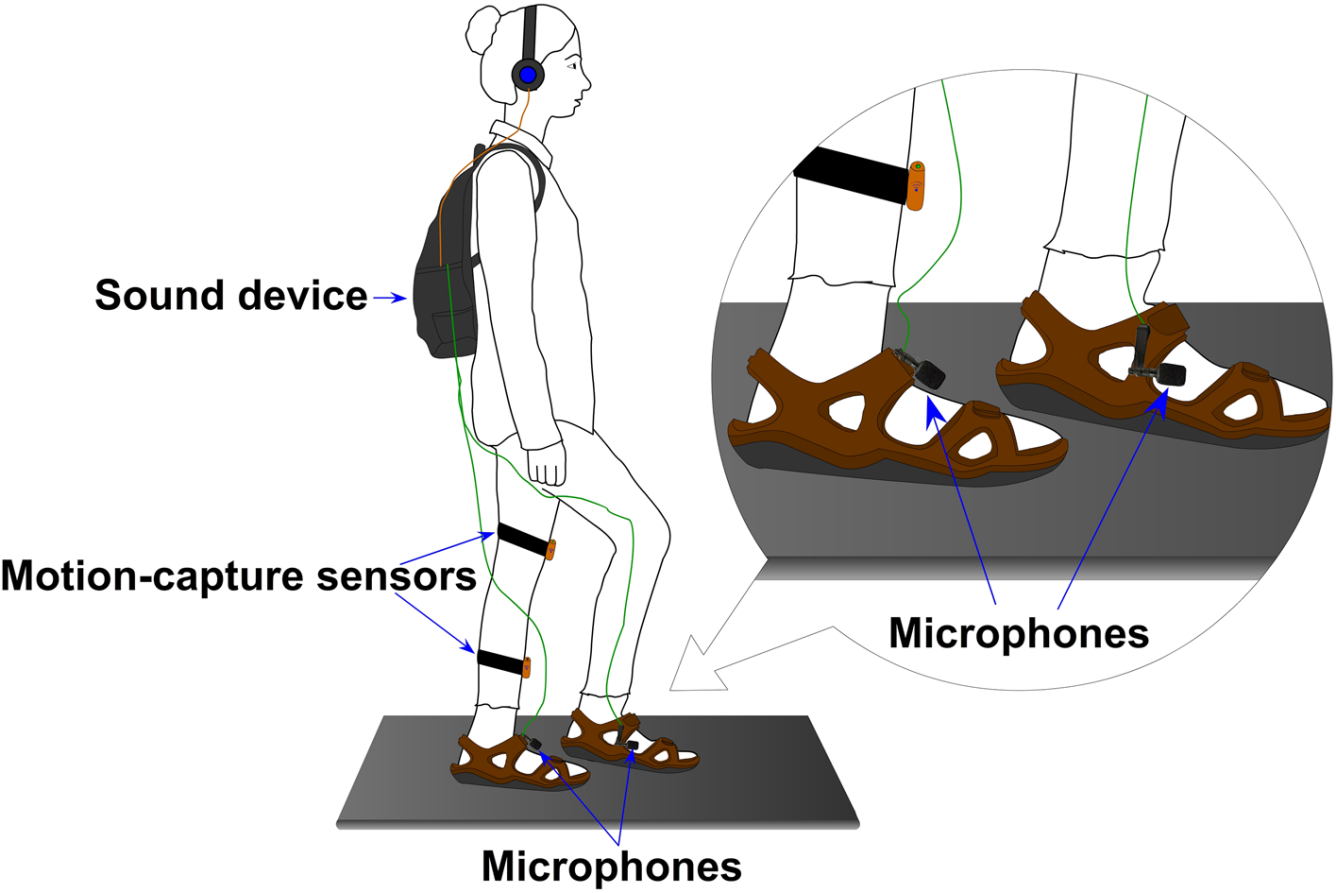
Overview of the shoe-based sound device and sensors used for sensing gait. The copyright holder of this image is the first author.

A shoe-based sound device, already used in related studies^40,48–52^, allowed the dynamic modification of footstep sounds, as people walk. The system (see Fig. 1) is comprised of a pair of strap sandals with hard rubber sole; two microphones attached to the sandals that capture the walking sounds (Core Sound); and a small stereo pre-amplifier (SP-24B) and a stereo 9-band graphic equalizer (Behringer FBQ800) that amplified the sounds and changed the sound spectra.

Three auditory feedback conditions were created by dynamically modifying the footstep sounds people produce as they walk, and which were captured by the microphones: a “High Frequency” condition, in which the frequency components of the footsteps sounds in the range 1–4 kHz were amplified by 12 dB and those in the range 83–250 Hz attenuated by 12 dB; a “Low Frequency” condition, in which the frequency components in the range 83–250 Hz were amplified by 12 dB and those above 1 kHz attenuated by 12 dB; and a “Control” condition, in which participants heard their natural footsteps sounds equally amplified across frequency bands. These were the same three conditions used in previous studies^40,50,52^ (see also the study by Li et al.^39^ in which the spectral components of waking sounds produced by others were manipulated similarly to influence the perception of the walker’s body characteristics).

The resulting sound was fed back via closed headphones (Sennheiser HDA300) with high passive ambient noise attenuation (> 30 dBA) that muffled the actual sound of footsteps. The analogue sound loop had minimal latency (< 1 ms). Pre-amplifier and equalizer were fitted into a small backpack the walker could carry (~ 2 kg, 35 × 29x10 cm). In addition, two wearable motion-capture sensors (www.wearnotch.com), placed on the left calf and thigh of participants, were used to collect gait-related position data, as in the study by Brianza et al.^48^.

The experiment was conducted in a quiet laboratory room. A wooden board (89 × 45x1 cm) was placed on the floor for participants to step on it. The ground and footwear materials are relevant as they affect the resulting sounds^39,40,65^. The hard rubber soles in contact with the wooden board produce clear sounds. A computer was placed near participants to collect their body estimates after each walking period (see below).

#### Experimental design

The experiment followed a repeated measures mixed design with two independent variables (IV) with 3 levels each: a within-subjects repeated-measures IV-auditory feedback condition—and a between-subjects IV-SED group. The three levels of auditory feedback condition corresponded to the “High Frequency”, “Low Frequency” and “Control” conditions. The three levels of SED were Low-, Medium- and High-SED. The order of the conditions, including the “Control” condition, was randomised.

To account for individual differences in disordered eating and in baseline body-representation distortions within each SED group^25^ we included as covariates, respectively, two measures. The first measure was the participant’s EDE-Q score, which relates to the participants’ subjective bodily feelings and attitudes towards their body. Note that even if the EDE-Q score was used to define the SED groups, by including this score as covariate we acknowledge the importance of considering the variation between participants within each group (see ranges in the participants section). While this could be considered as a potential confound, it can be considered valid in the context of exploration of a data set, as in the case of this pilot study, as a means of understanding the patterns of variance in the data set^66^. The second measure was the participant’s baseline distortion in the body size visualization (to which we refer to as body image distortion), which was measured by calculating the weight differential between the actual body weight of participants and their estimated weight when using a body-visualization tool (i.e., Body Visualizer, see below and Table S1 in Supplementary material with a summary of the weight differential measure taken at baseline for all SED groups). We did not include BMI as covariate to avoid multicollinearity, as BMI strongly correlated with the other two covariates (e.g., with EDE-Q, r = 0.51, *p* < 0.001).

#### Outcome measures

*1. Body-Visualization* The tool “Body Visualizer” (bodyvisualizer.com) was employed to collect participants’ visual estimates of their own body weight, in keeping with previous studies^40,51,52,67^. Participants observed a 3D female avatar on the computer screen. The proportional ‘height’ of the avatar corresponded to participants’ actual height, and it was kept constant throughout the experimental session. The initial ‘weight’ of the avatar was set to match the participant’s weight (that we measured at the beginning of the testing session) ± 25%. Participants were asked to adjust the ‘weight’ dimension of the avatar’s body by using the left and right arrows of the keyboard to correspond to their own perceived body size (see similar procedures^67–69^). Whether the initial weight was + 25% or -25% was counterbalanced across two repetitions, performed one after another, and which together allowed calculating the body-visualization measure for each condition, by averaging the two responses. Note that, as in the case of condition randomization, varying the initial avatar’s weight across trials allowed to compensate for practice bias, as it avoided anchor effects of the initial value. It should also be noted that participants could only see the 3D avatar on the computer screen, as the numerical values of the weight and other body parameters were hidden from their view and were only accessible to the experimenter.

Baseline measures of body image distortions^26^ were also collected (i.e., pre-experimental task differences between perceived and actual body weight using the Body-Visualization task) to understand whether these distortions are larger in our target groups (i.e., High-SED and AN). If such baseline distortions do exist, then they need to be considered as a potential modulating factor of the changes in distortions induced by the body-weight illusion, specifically in terms of the affective bias experienced towards the change (e.g., to perceive a ‘lighter body’ as a positive change vs. a ‘heavier body’ as a negative change).

**Figure 2:**
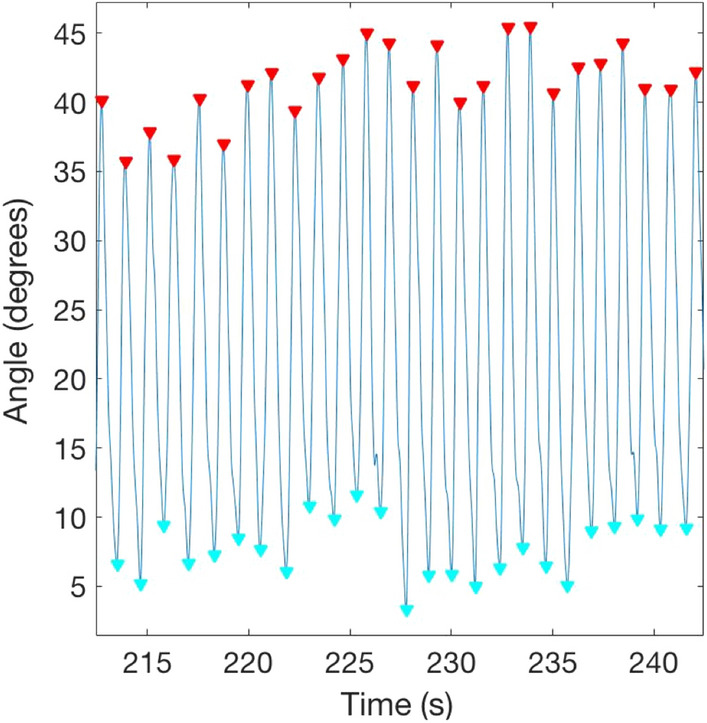
Gait data on left leg knee sensor—red triangles mark the maximum knee angle and blue triangles mark the minimum angle in each gait cycle; from these values and times one can derive the knee acceleration, stride time and gait speed in each condition. This measure provided behavioural information about implicit distortions of body weight/representation.

*2. Gait biomechanics* These were used as an implicit measure of changes in perceived body weight, as in previous studies^40,48,52^. Gait patterns of people with lighter bodies are characterized by larger leg accelerations^53,54^, while those of people with heavier bodies are characterized by slower gait speed and longer durations of the stance and the heel strike^53^. Previous studies have shown that the auditory-driven illusion of having lighter/heavier bodies resulted in people adapting their gait to match the prototypical gait pattern of lighter/heavier bodies^40,48,52^. As in the study by Tajadura-Jiménez et al.^40^, we focused on data from the left leg since we did not have any specific hypothesis on left/right asymmetries and due to sensor availability. We quantified the knee lifting acceleration, and the stride time (time between two touchdown events, from which cadence – i.e., gait velocity in steps/minute—can be derived) of each gait cycle (i.e., the time between two successive steps made by one foot)^70^ (see Fig. 2). Gait patterns were operationalized with these two parameters: lifting acceleration and stride time.

*3. Questionnaire on emotional state* We recorded valence (unhappy—happy), arousal (calm—excited) and dominance (submissive—dominant) felt by participants at baseline and the end of each walking condition, using the 9-item graphic scales of the self-assessment manikin^71^.

*4. Questionnaire on body feelings* Participants were asked to select a score that best expresses their feelings at baseline and during each sound condition using 7-point Likert-type response items. The questionnaire, which was adopted from the study by Tajadura-Jiménez et al.^40^, comprised four statements which ranged from: “I felt slow” to “I felt quick” (Speed); “I felt light” to “I felt heavy” (Weight); “I felt weak” to “I felt strong” (Strength); and “I felt crouched, stooped” to “I felt elongated, extended” (Straightness); and four statements for which participants were asked to rate their level of agreement (from strongly disagree to strongly agree). The first of these four statements checked whether participants felt as if they were the agents of the sounds (Agency), as many studies have shown that large discrepancies between modalities and delays between actions and sensory feedback disrupt agency and diminish the sensory-induced bodily illusions^45,72^. Two statements checked whether participants had a vivid feeling (“It seems the feeling of my body is less vivid than normal”; Vividness) or had surprising and unexpected feelings about their body (“The feelings about my body are surprising and unexpected”; Surprise). Finally, we also looked at foot localization (“It seems like I could really tell where my feet are”) as sound may interact with proprioception^45,72^.

#### Procedure

**Figure 3:**
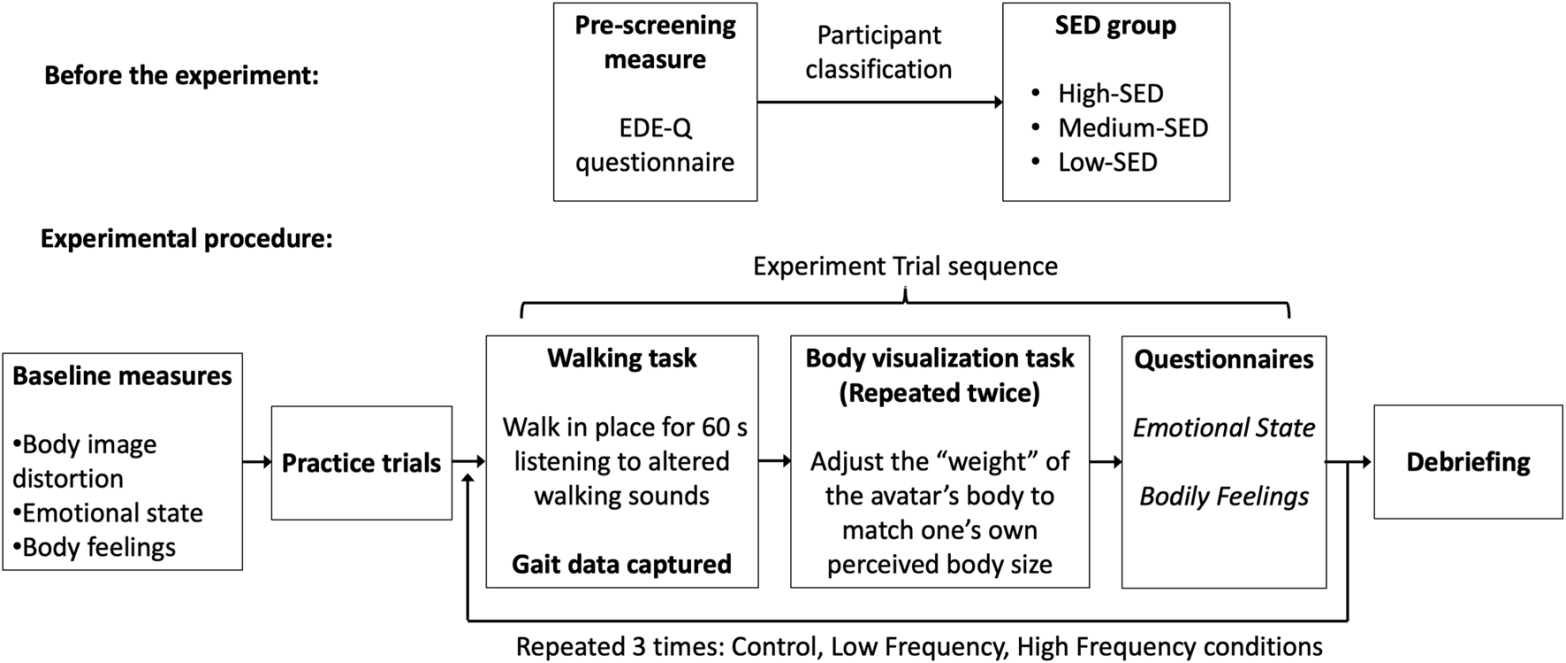
Graphical representation of the experimental procedure.

Figure 3 shows a graphical representation of the experimental procedure. Prior to the experiment, participants completed the EDE-Q questionnaire (presented in online format). Participants eligible to take part were invited to the lab. After reading the information sheet and signing the consent form, participants were equipped with the shoe-based sound device and the motion-capture sensors. For hygienic purposes, each participant was provided with freshly cleaned socks and only these were allowed to be in contact with the shoes. They were then introduced to the body-visualization task, and they were asked to adjust twice the avatar using the Body Visualizer (the initial avatar weight varied, + 25% or −25%, in randomized order) to obtain a baseline measure of participant’s body image distortion (i.e., the weight differential) prior to being exposed to the experimental manipulations. Participants also completed the questionnaires on emotional state and body feelings to collect baseline data on those measures.

Then, participants were instructed in the walking task and were asked to complete three practice trials (Low Frequency, High Frequency and Control, each lasting 60 s) in a randomized order. This was done to allow participants to familiarise themselves with the experimental procedure and to avoid any surprise effects due to the unusual sounds that might have interfered with the task performance. All the conditions were used as practice trials to avoid any bias for one condition or another. Then, participants completed three experimental trials that were presented in another randomized order. In each trial, participants were asked to walk in place at a self-paced, comfortable speed for 60 s and on the wooden board on the floor, while wearing the shoe-based sound device and the motion-capture sensors (see Fig. 1). After each walking condition, participants completed the body-visualization task and the questionnaires on body feelings and emotional state. Participants were not given any information or feedback about the corresponding height or weight of the avatar, or about their performance on the other tasks, at any point during the experiment. Note that the “body image distortion” baseline measure and the “body-visualization” outcome measure after each walking condition were actually obtained using the same method (i.e., using the tool “Body Visualizer”), but they were obtained at different times during the experimental procedure. Namely, the body image distortion at baseline was obtained only once, before any experimental manipulation, while the variable referred to as “body visualization” is the outcome measure of our auditory experimental manipulation. As such, we obtained multiple body visualizations measurements that we can compare across experimental conditions, while we only have one body image distortion measurement. The full procedure took 50 min. Following completion of the experiment, participants were fully debriefed.

#### Data analyses

Body-Visualization and gait data were statistically analysed with SPSS 25.0. Shapiro–Wilk tests assessed normality of data distributions. For the baseline data, we conducted a one factor ANOVA, to compare the mean body image distortion between SED groups, followed by *t*-tests comparing the three groups against each other to understand if there were differences in body image distortions, and applying Bonferroni corrections for multiple comparisons. For the experimental conditions, the Body-Visualization data were LOG-transformed to improve the distribution, as in previous studies^40^. Raw sensor (gait) data were extracted using MATLAB software. For all steps in each condition, we calculated the average leg-up and leg-down acceleration; the stride time and the number of steps/minute (cadence). As in related studies^40,50^, individual z-scores were calculated for the gait data to improve the distribution and reduce intra-subject variability^73^. Body Visualization and gait data were then analysed with repeated measures analyses of covariance (ANCOVA), with sound condition (‘Control’, ‘High frequency’ and ‘Low frequency’) as within-subject factor, SED group as a between-subjects factor, and the EDE-Q score and body image distortion as covariates. Significant effects were followed by paired samples *t*-tests, with the significance alpha level adjusted for multiple comparisons using Bonferroni correction.

For questionnaire data, we conducted non-parametric ANOVAs on aligned rank transform (ART) data using R software. The ART relies on a pre-processing step that “aligns” data before applying averaged ranks, after which common Analyses of Variance (ANOVA) can be applied. For the baseline data, we used the R package ARTool^74^ to compare the questionnaire scores between SED groups. For the experimental conditions, we conducted non-parametric ANCOVAs on ART data using the R package ‘npsm’^75^, with sound condition (‘Control’, ‘High frequency’ and ‘Low frequency’) as within-subject factor, SED group as a between-subjects factor, and the baseline EDE-Q score and body image distortion as covariates. In case of significant main effects of sound condition or of SED group, these were followed by *t*-tests on ART data comparing the three conditions or the three groups against each other, with the *p*-value adjusted with the recommended Tukey method for comparing a family of 3 estimates^74^.

### Experiment 2: Effects of altered auditory feedback in clinical population with AN

#### Participants

Fifteen women (Mean age ± s.d.: 26.53 ± 9.8 years; age range: 18–49 years), naïve to the study aim, were recruited from the inpatient’s unit at the ITA-ABB Center (“Centro de Prevención y Tratamiento de Anorexia y Bulimia”) in Seville, Spain. The sample size was based on availability of participants at the center complying with the inclusion criteria during the study period. All patients met the Diagnostic and Statistical Manual of Mental Disorders, 5^th^ Edition (DSM-V^5^) criteria for AN, as assessed using the Structured Clinical Interview for DSM-IV Axis I Disorders (SCID). Exclusion criteria included having bulimia nervosa or any other specified and unspecified eating disorders, a BMI above 18.5, being a male and any substance abuse (i.e., drug and alcohol). Our sample had an average BMI of 17.17 (± 1.03, range 14.39–18.32) suggesting overall moderate to mild BMI consequences in the context of prior AN research^76^; however, BMI was not formally used as an index of illness severity given well-documented problems with this measure^77^. Instead, we focused on distortions in body-visualization as an index of severity of the specific ED feature, namely body image distortions (as quantified with the variable “weight differential”), that most relate to our hypotheses about body-representation. Following a consultation with the clinical team at the ED centre, we did not collect the EDE-Q score for this sample as asking participants to fill in this questionnaire was considered a sensible and potentially upsetting procedure (see supplementary material for other clinical details on the recruited participant sample).

All participants provided informed consent prior to their inclusion in the studies. The experiment was conducted in accordance with the ethical standards laid down in the 1964 Declaration of Helsinki, as revised in 2008, and approved by the corresponding ethics committee of *Junta de Andalucía*.

#### Materials

Experiment 2 used the same shoe-based sound device employed in Experiment 1 (see Fig. 1). Due to availability, to measure gait we used a different system, which has been successfully employed in other studies^50,52^. This system consists of four force sensitive resistors (FSR) attached to the sandal insoles; and two 9-axis MotionTracking devices (MEMS) placed on the participant’s ankles. FSRs and MEMS in each foot connect to a Microduino board attached to the sandals, which links the sensors via Bluetooth to a smartphone which captures the data with an especially developed app.

The experiment was conducted in a quiet room. This room had a hard marble floor, and therefore it was optimal for the study as the hard rubber soles in contact with the marble floor produce clear sounds. A computer was placed near participants to collect their body estimates after each walking period.

#### Experimental design

The experiment followed a repeated-measures design with a within-subjects repeated-measures IV—auditory feedback condition—with three levels that correspond to the same three auditory feedback conditions applied in Experiment 1: “High Frequency”, “Low Frequency” and “Control” conditions. The same measures applied in Experiment 1 to assess changes in body-visualization, gait patterns and body feelings and emotional state (questionnaire data), were used. In Experiment 2 there was a single group of participants, to which we refer to as AN group. We report the within-group differences between conditions for this AN group, as well as compare the between-groups effects across the participants in Experiments 1 and 2.

#### Procedure

The same procedure as in Experiment 1 was followed (see Fig. 3).

#### Data analyses

Body-Visualization data and gait data were statistically analysed with SPSS 25.0. Raw sensor data (gait) were extracted using MATLAB software—for all steps in each condition, we calculated the average leg acceleration and the stride time.

Shapiro–Wilk tests assessed normality of data distributions. The Body-Visualization data were normal, but the gait data were not. Therefore, as in Experiment 1 and in previous studies^40^, individual z-scores were calculated to improve the distribution of the gait data and reduce intra-subject variability^73^. Body-Visualization and gait data were then analyzed with one-way repeated measures ANCOVA, with sound condition (‘Control’, ‘High frequency’ and ‘Low frequency’) as within-subject factor. To account for individual differences in the severity of the condition related to body image distortions, we included as covariate the baseline body image distortion as in Experiment 1. Significant effects were followed by paired samples one-tailed *t*-tests comparing the three sound conditions against each other, given that we had a strong unidirectional hypothesis for the possible differences between the three conditions; the significance alpha level was adjusted for multiple comparisons using Bonferroni correction (significance alpha = 0.033).

For questionnaire data, we conducted non-parametric ANCOVAs on ART data using the R package ‘npsm’^75^, with sound condition (‘Control’, ‘High frequency’ and ‘Low frequency’) as within-subject factor and the baseline body image distortion as covariate. In case of a significant main effect of sound condition, this was followed by t-tests on ART data, comparing the three sound conditions against each other and with the *p*-value adjusted with the recommended Tukey method for comparing a family of 3 estimates^74^.

## Results

### Experiment 1: Effects of altered auditory feedback in healthy individuals with low, medium and high symptomatology of ED

#### Baseline measures: Body weight distortion, emotional state and body feelings

A summary of the baseline measures for the three SED groups is presented in Supplementary material (see Supplementary Table S2). In brief, our results show that all SED groups underestimated their body weight in the Body-Visualization task. The weight differential (our index of *body image distortion*) is larger for the High-SED compared to the Low-SED group, but this difference did not reach significance. Given the large SD, we considered it important to include this body image distortion at baseline as a covariate in subsequent analysis to control for its potential effect on the way participants experienced the body-weight illusion. In terms of body feelings and emotional state at baseline, the High-SED group reported feeling significantly heavier and less happy than the Low-SED group, and they felt overall less agency over their produced walking sounds than the Medium-SED group. These results point towards an increased negativity and altered body sensations in the High-SED group. Correlation analyses between the variable happiness and the participants’ EDE-Q scores (global and scales) confirmed the hypothesis that negative emotions towards their body are already present before the experiment in the participants scoring high in the EDE-Q shape and weight concern scales (as well as in the EDE-Q global score), as results showed that, indeed, the more concerned participants were with their shape and weight, the less happy they were.

#### Effect of sound condition on body-visualization according to SED

**Figure 4:**
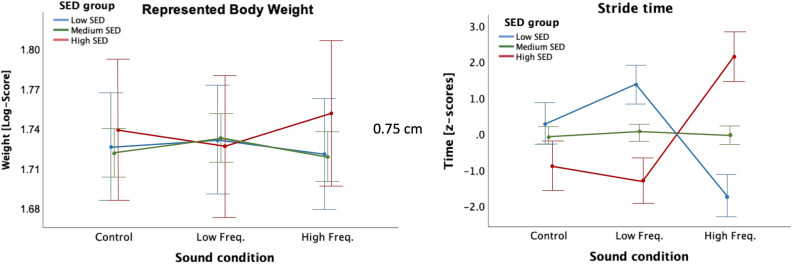
(left) Mean(± SE) represented body weight (LOG-Scores) for all sound conditions and for each SED group in Experiment 1, with EDE-Q global score and baseline body image distortion (difference between represented weight at baseline and actual weight) as covariates. (right) Mean(± SE) stride time (z-scores) for all sound conditions and for each SED group in Experiment 1, with EDE-Q global score and baseline body image distortion as covariates.

The ANCOVA on the LOG-transformed Body-Visualization data showed a no significant, interaction between sound condition and SED group (F(4,106) = 2.34, *p* = 0.060, eta2 = 0.081). As shown in Fig. 4 Left, participants in the Medium-SED group, and to a lesser extent in the Low-SED group, represented their body as bigger in the Low Frequency condition compared to the other two conditions, and showed a smaller represented weight in the High Frequency condition. However, the pattern seems inverted for the High-SED participants, who showed a smaller represented body weight in the Low Frequency condition and a larger represented body weight in the High Frequency condition. No main effect of sound condition or SED group, nor significant interactions with EDE-Q global score and weight differential were found (all ps > 0.4).

#### Effect of sound condition on gait patterns

Due to technical issues, gait data was correctly recorded for only 33 participants (10 Low-SED, 10 High-SED, 13 Medium-SED). We tested our hypotheses with these data. As specified in the “Data Analysis” section, Z-scores for each gait variable were submitted to an ANCOVA with the within-subject factor sound condition, with SED group as a between-subjects group and with covariates the EDE-Q global score and body image distortion.

The ANCOVA on the stride time data showed a significant main effect of sound condition (F(2,54) = 7.05, *p* = 0.002, eta2 = 0.207), and interaction of sound condition with SED group (F(4,54) = 3.11, *p* = 0.022, eta2 = 0.187) and with EDE-Q global score (F(2,54) = 6.61, *p* = 0.003, eta2 = 0.197). Post-hoc analysis on the main effect of sound showed overall non-significant differences between sound conditions (Mean(SD): Control: −0.20(0.14); Low Frequency: 0.06(0.13); High Frequency: 0.14 (0.14)). With respect to the interaction of sound condition with SED group, we conducted one-way ANCOVAs separately for each sound condition, with the between-subject factor SED group and with the individual EDE-Q global score and body image distortion as covariates. For the High Frequency condition, there was a significant difference between SED groups (F(2,27) = 5.17, *p* = 0.013, eta2 = 0.277); post-hoc paired comparisons between sound SED groups showed significant differences between all groups: High vs. Low SED (*p* = 0.012), High vs. Medium SED (*p* = 0.039) and Medium vs. Low SED (*p* = 0.016). For the Low Frequency condition, there was also a significant difference between SED groups (F(2,27) = 3.56, *p* = 0.042, eta2 = 0.209); post-hoc paired comparisons between SED groups showed significant differences only between Medium and Low SED groups (*p* = 0.039). There were no significant differences between SED groups for the Control condition (*p* = 0.58) (see Fig. 4 Right).

For acceleration, results showed no significant main effect of sound condition (F(2,56) = 3.03, *p* = 0.056, eta2 = 0.098) and interaction of sound condition with SED group (F(4,56) = 2.07, *p* = 0.093, eta2 = 0.130). Being acceleration and stride time related parameters, we do not expand on this analysis here, but this is included as supplementary material (see Supplementary Fig. S2).

#### Effect of sound condition on body feelings and emotional state

**Figure 5:**
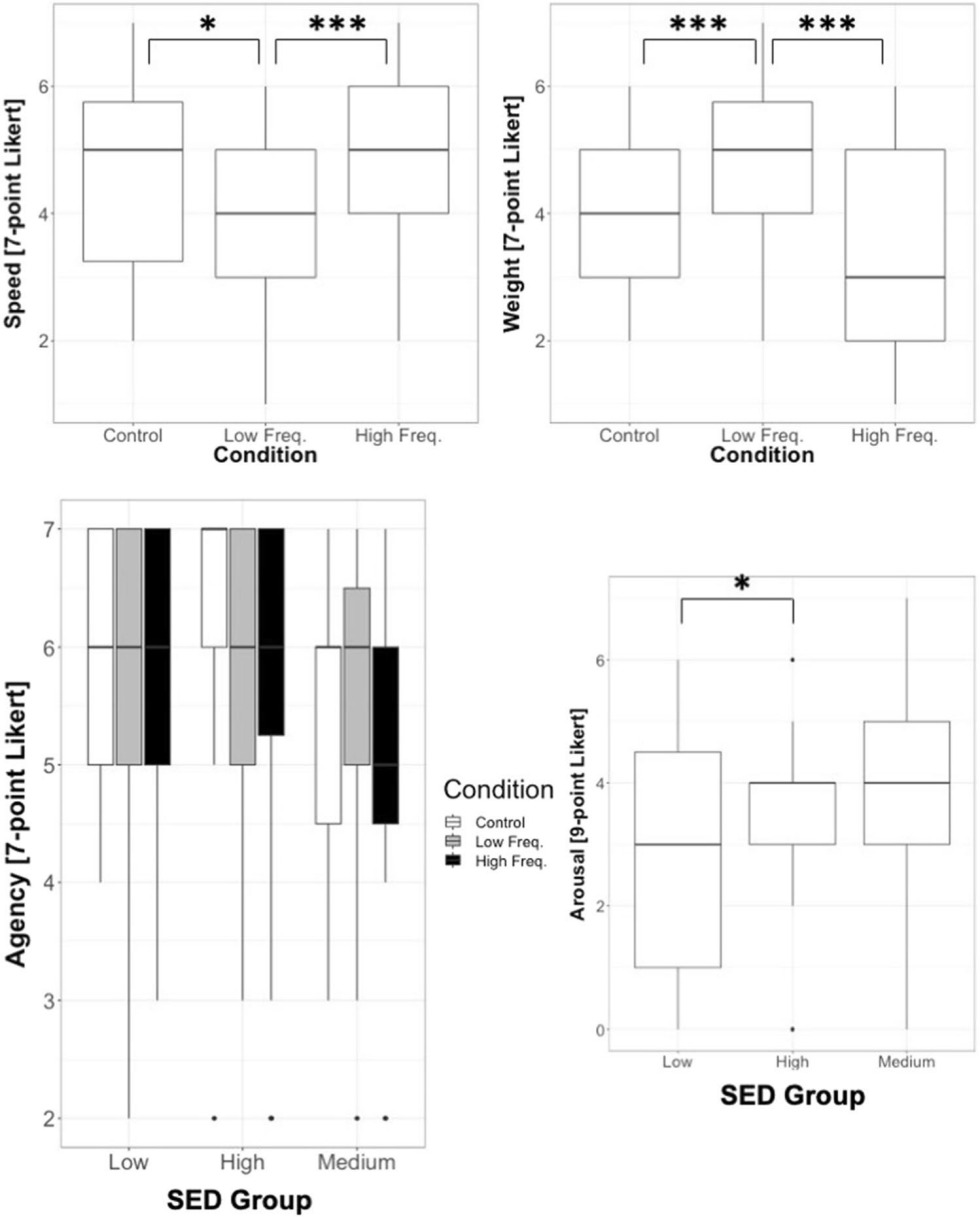
Median (± Range) for questionnaire items for which there were significant effects of Sound in Experiment 1 (HF-High Frequency, LF-Low Frequency, C-Control) or of SED group (Low-, Medium-, High-SED).

Supplementary Table S3 shows the Median (Range) for all items for the three groups of participants (see also Supplementary Table S4 and more details on the analyses performed in Supplementary Materials. In brief, the analyses revealed significant effects for Speed, Weight, Agency and Arousal. For Speed and Weight, there was only a significant main effect of sound condition (Speed: F(2,110) = 5.08, *p* = 0.007; Weight: F(2,110) = 10.10, *p* < 0.001), which was in line with the effects observed in previous studies^40,52^, but there were no effects related to Group differences that supported our hypothesis. As shown in Fig. 5, participants felt overall quicker and lighter in the Control than in the Low Frequency condition and in the High Frequency than in the Low Frequency condition, with no significant difference between the High Frequency and the Control conditions. For Agency, there were significant main effects of sound condition (F(2,110) = 6.98, *p* = 0.001) and of SED group (F(2,55) = 6.98, *p* = 0.001), as well as a significant interaction between both factors (F(4,110) = 3.49, *p* = 0.009), but follow up comparisons did not reach significance. In Fig. 5 the significant interaction between sound condition and SED group for the agency score can be observed. For Arousal, there was only a significant main effect of the SED group (F(2,55) = 10.69, *p* < 0.001) which is shown in Fig. 5, but follow up comparisons between Groups did not reach significance.

In addition to the above reported analysis, we ran the same analysis without EDE-Q global scores as a covariate. While for the questionnaire results there were only small changes, we also found that there were non-significant effects of sound manipulation in the other variables of interests (i.e., body visualization and gait patterns). This might suggest the relevance of taking into account individual differences in SED within each group when studying body weight distortions in auditory-driven body illusions (see supplementary material for more details).

### Experiment 2: Effects of altered auditory feedback in clinical population with AN

#### Baseline measures: body weight distortion, emotional state and body feelings

As in Experiment 1, we collected a baseline measure of distortions of body-representation to understand whether these distortions are present in our target group (i.e., AN). A summary of this measure and of the emotional state and body feelings of AN participants also measured at baseline is presented in Supplementary material, together with a comparison with the results for the groups in Experiment 1. In brief, our results show that participants do not show large distortions in their perceived body weight, as compared to their actual weight (as suggested in the study by Cornelissen et al.^25^. Further, at baseline AN participants as a group felt slightly slow, heavy, weak and crouched; they also felt neutral in terms of happiness and arousal, and slightly not in control.

#### Effect of sound condition on body-visualization

**Figure 6:**
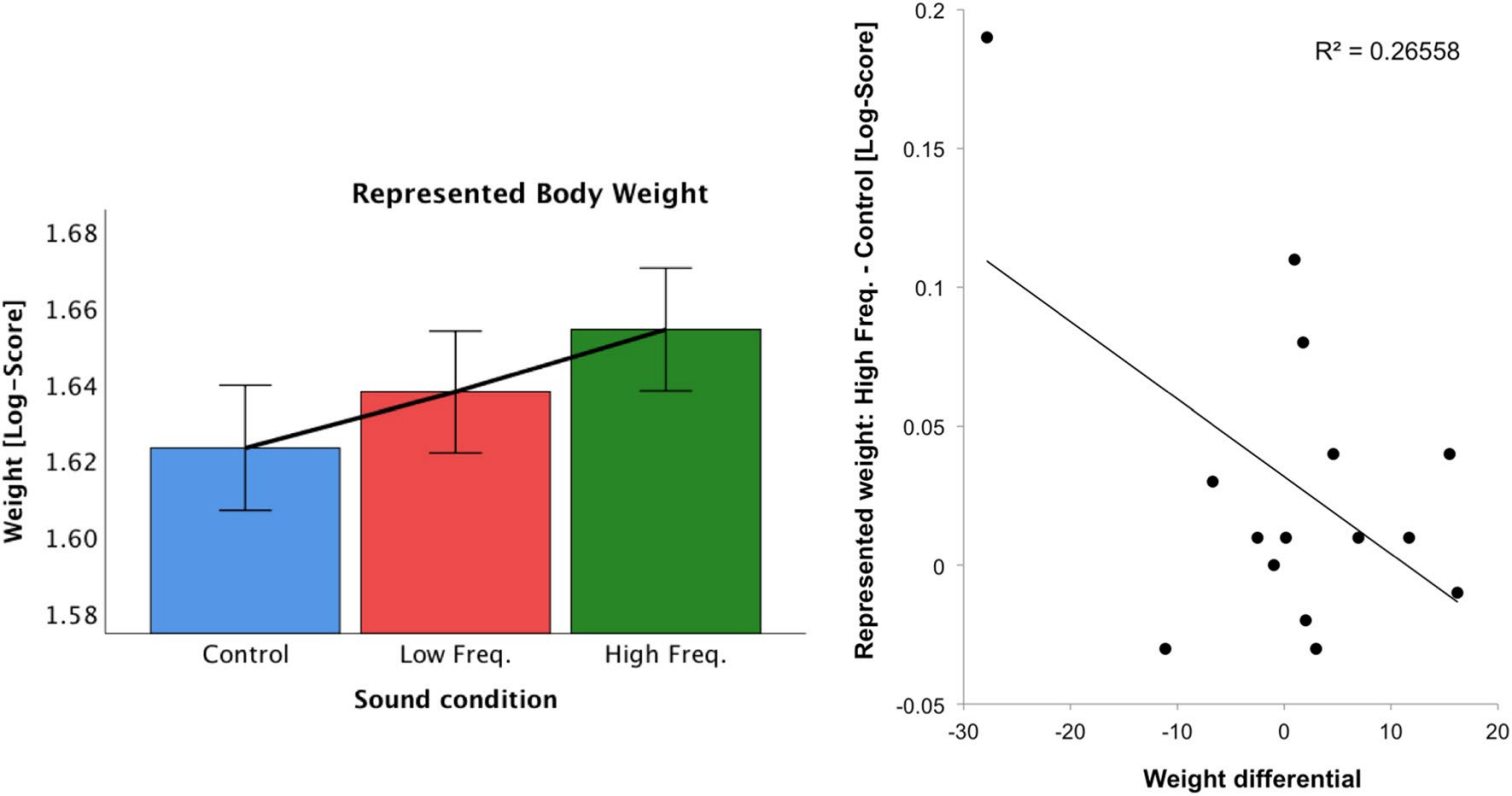
(left) Mean(± SE) represented body weight (LOG-Scores) for all sound conditions in Experiment 2, with weight differential (difference between represented weight at baseline and actual weight, our index of body image distortion at baseline) as covariate. (right) Correlations between Weight differential and the difference in represented body weight between High Frequency and Control conditions (LOG-scores) in Experiment 2.

Results from Experiment 1 suggest that in High-SED participants, contrary to our predictions, the High Frequency condition led to a slimmer/lighter represented body, while the Low Frequency condition led to a wider/heavier represented body, as compared to the Control condition (no-sound frequency manipulation). To confirm and expand these results with the data from AN participants, and as specified in the “Data Analysis” section, we ran an ANCOVA with a within-subject factor sound condition and with the body image distortion as covariate. The ANCOVA showed a significant main effect of sound condition (*F*(2,26) = 3.42, *p* = 0.048, eta2 = 0.208) and a significant interaction of sound condition with the body image distortion (*F*(2,26) = 6.46 *p* = 0.005, eta2 = 0.332). Follow-up paired comparisons for each condition against each other revealed a higher represented body weight in the High Frequency than in the Control condition (*p* = 0.040), although this result does not survive multiple comparisons (See Fig. 6 Left).

To further investigate the interaction between sound condition and body image distortion, we calculated the difference between the High Frequency and Control conditions, and between the Low Frequency and Control conditions and correlated these two variables with the body image distortion (see Fig. 6 Right). Our results show a negative correlation between the body image distortion and the difference in represented body weight between High Frequency and Control conditions (r = 0.52, *p* = 0.047)—people with a negative weight differential or baseline body image distortion (i.e., those that perceived their body as wider/heavier than their actual body) represented their body as heavier in the High Frequency than in the Control Condition.

**Figure 7:**
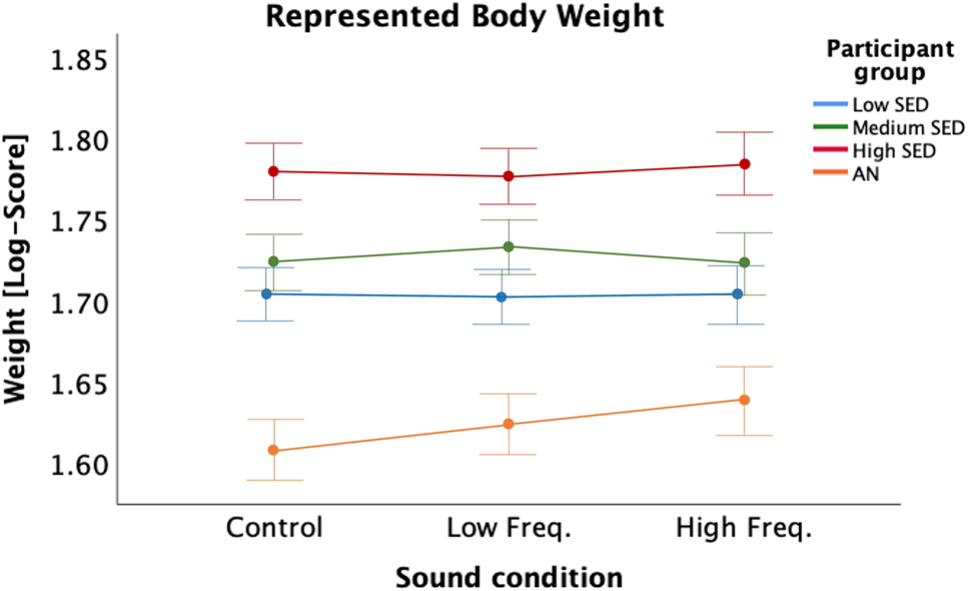
Mean(± SE) represented body weight (LOG-Scores) for all sound conditions and for each Participant group in Experiments 1 and 2 (Low-SED, Medium-SED, High-SED, AN), with baseline body image distortion (difference between represented weight at baseline and actual weight) as covariate.

To understand whether the effects found were significantly different to those observed for the groups in Experiment 1, we conducted an ANCOVA, with sound condition as within-subject factor and participant group as between-subjects factor, and body image distortion as covariate. The ANCOVA on the LOG-transformed data showed no significant effects of sound condition (*F*(2,136) = 2.95, *p* = 0.056, eta2 = 0.042) and an interaction of sound condition with participant group (*F*(6,136) = 2.095, *p* = 0.058, eta2 = 0.085). The interaction with the body image distortion did not reach significance (*p* > 0.14). As shown in Fig. 7, the Medium-SED participants represented their body as heavier in the Low Frequency condition than in the other two conditions, with a smaller represented weight in the High Frequency condition, but the pattern seems inverted for the High-SED participants. For AN participants it is even more obvious that the represented weight in the High Frequency condition is larger than in the Control Condition, with the represented weight in the Low Frequency condition falling between the one in the other two conditions.

#### Effect of sound condition on gait patterns

**Figure 8:**
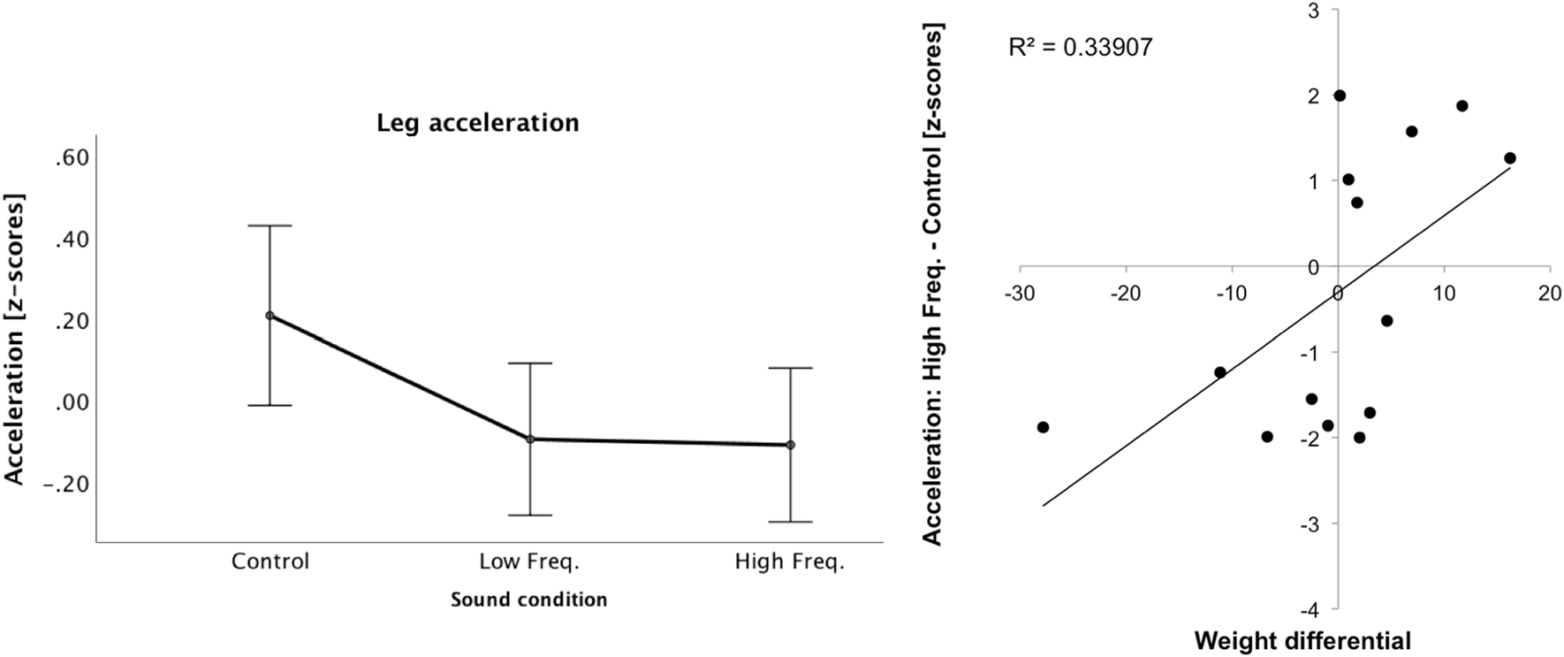
(left) Mean(± SE) foot acceleration (z-scores) for all sound conditions in Experiment 2, with body image distortion as covariate. (right) Correlations between Weight differential (our index of body image distortion at baseline) and the difference in foot downwards acceleration between High Frequency and Control conditions (z-scores) in Experiment 2.

Results from Experiment 1 showed a pattern of results for the High-SED participants that was opposed to our prediction that in the High Frequency condition participants will adopt a behaviour consistent with having a “lighter” body, characterized by more accelerated swings and less foot–ground contact time. In Experiment 2, due to technical issues, gait data was not recorded for one participant. Our analyses focused on confirming and expanding the results from Experiment 1 with data from the remaining 14 participants with AN. As specified in the “Data Analysis” section, Z-scores for each gait variable were submitted to an ANCOVA with the within-subject factor sound condition, and with the baseline body image distortion l as covariate. We found a significant interaction effect between sound condition and the body image distortion for the foot acceleration in the downwards movement (F(2,24) = 4.32, *p* = 0.025, eta2 = 0.265). While the main effect of sound was not significant (*p* = 0.6), the interaction between sound condition and body image distortion suggest that the altered sound conditions (High and Low Frequency) resulted in lower acceleration compared to the Control condition (see Fig. 8 Left).

To further investigate the interaction of sound condition with the body image distortion, we calculated the difference between the High Frequency and Control conditions, and between the Low Frequency and Control conditions, in foot downwards acceleration and correlated these two variables with the weight differential or body image distortion at baseline (see Fig. 8 Right). Our results showed a significant positive correlation between the baseline body image distortion and the difference in acceleration between High Frequency and Control conditions (*r* = 0.58, *p* = 0.029)—people with a negative weight differential or baseline body image distortion(i.e., those that perceived their body as wider/heavier than their actual body) showed less foot acceleration in the High Frequency than in the Control Condition.

#### Effect of sound condition on body feelings and emotional state (questionnaire data)

The analyses performed on data from AN participants did not reveal an effect of sound condition (see median scores for questionnaire items and details on the analyses performed in Supplementary material and Table S7). Nevertheless, it was found that larger weight differentials predicted lower reported strength (F(2,7.65) = 12.55, *p* = 0.001) and higher reported agency (F(2,8.16) = 13.57, *p* < 0.001) (see Fig. S4).

Results on the analyses performed comparing the four groups of participants did not support the hypothesis of enhanced effects for the AN group, as compared to the different SED groups in Experiment 1. While we replicated previous results in healthy participants with regards to effects of sound condition in Speed (F(2,2.49) = 3.81, *p* = 0.023) and Weight (F(2,7.86) = 12.71, *p* < 0.001), as participants felt overall quicker and lighter in the High than in the Low Frequency condition, we also found a significant interaction between sound condition and participant group (High-SED, Medium-SED, Low-SED, AN) both for Speed (F(6,5.19) = 2.64, *p* = 0.017) and Weight (F(6,5.53) = 2.98, *p* = 0.008). This was mainly caused by the fact that, contrary to our hypothesis, in the High Frequency condition AN participants felt heavier and slower than in the Control condition, although follow-up comparisons did not reach significance (see full analysis in Supplementary material and Fig. S4). We also found significant effects of the SED group for several items, independently of the effect of sound condition, although follow-up comparisons did not reach significance (see full analyses comparing the four groups of participants in Supplementary material).

## Discussion

Across two experiments, we aimed to investigate whether people with heightened subclinical SED (in Experiment 1) and clinical population with AN (in Experiment 2) show an enhanced susceptibility to an auditory-driven body weightily illusion compared to those with Low- or Medium-SED. To address our experimental question, we focused on the way participants visually represented their body (*body-visualization tool*), on their body movements (*gait biomechanics)* consistent with having a lighter or heavier body, and on their body feelings and emotional states (*self-report questionnaire*), in response to our experimental manipulation.

Our results confirmed differences in how sound can modulate body-representation, as related to the perceptual component of body image, according to ED symptomatology, although these differences were not in the expected direction. Specifically, we found that people with High-SED and people with AN, perceive their body as wider/heavier and display the longest stride times in the ‘High-Frequency’ condition, which typically leads to a reverse type of behaviours (i.e., lighter perceived body and shorter stride times), as compared to the ‘Low-Frequency’ and ‘Control’ conditions. In contrast, people with Medium- and Low-SED showed a pattern of perceptual and attitudinal behaviours in line with previous studies conducted with healthy participants^40,49,52^ (i.e., the results are consistent with participants representing their body as lighter in the ‘High-Frequency’ condition and as heavier in the ‘Low-Frequency’ condition, as compared to the other two conditions), thus confirming the robustness of the footsteps illusion. As such, these results demonstrate an unusual influence of exteroceptive auditory cues in the body-representation of people with High-SED and AN. Critically, these results were not consistent with the hypothesis of an overreliance on exteroception^35^, but they rather suggest a disturbance in the process of integrating the bodily information signalled by the auditory cues into one’s perceptual body image, as assessed by the body-visualization and gait biomechanics measures. This is consistent with previous findings highlighting sensory integration abnormalities in AN, whereby people with AN fail to weight their perceptual beliefs related to body perception against sensory evidence (e.g., size-weight illusion^78^; but see^79,80^). Based on our results, it is not possible to disentangle whether people with High-SED and AN show difficulties in the integration of exteroceptive signals only, or whether such disturbances also extend to the integration of such signals with other bodily signals (e.g., interoceptive and proprioceptive) to create a coherent affective, cognitive, and perceptual experience of their body (see review^34^). Nevertheless, these difficulties might potentially explain, at least in part, the body image distortions that have been traditionally reported in people with AN (in keeping with^14,36^).

In terms of the subjective, explicit measures of the study targeting attitudinal body image, we found that overall participants felt lighter and quicker in the High Frequency condition, in keeping with previous studies and regardless of group. Interestingly, the self-report measure collected at baseline showed a negative bias on felt weight and emotional state in the High-SED group as compared to the Low- and Medium-SED groups, as they reported feeling significantly heavier and less happy. This might suggest that negative emotions towards their bodily signals are already present before any experimental manipulation, and this might result in altered sensory processing. However, there was no significant interaction of SED group with sound condition on the self-report measures in Experiment 1, except for the agency component, where the High-SED group showed higher agency in the control condition, although this effect was non-significant (see related negative findings in recent work^81^). This dissociation between measures of body-representation changes, with self-report measures on the one hand assessing the attitudinal component of body image, and body visualization and gait on the other hand assessing the perceptual component of body image, is in line with other studies showing sensory effects for body-tracking measures but not for questionnaire measures^60^. It should be noted that, while the comparison between the AN group in Experiment 2 and the three groups of participants in Experiment 1 should be taken with caution, there was a significant interaction of group with sound condition for two of the questionnaire items, Weight and Speed. AN participants, as compared to the other participants, reported feeling heavier and slower in the High frequency than in the Control condition. This different pattern in AN might support the hypothesis of altered sensory integration processing in the AN group.

How can we interpret the unexpected finding that people with High-SED and AN perceive their body as heavier in the High-Frequency condition? We believe that our findings can be contextualised within a predictive coding framework, according to which perception depends on the ambiguity or noise of the sensory signals (i.e., precision weighting) that in turn influences the extent to which these signals are used to update predictions about the source of the stimulus to be perceived^82,83^. The brain is not a passive organ, but it acts as a predictive machine and it interprets sensory information with the main purpose of minimizing prediction errors (i.e., the difference between predictions and the actual signal). To this end, the brain forms Bayesian-optimal predictions, which must constantly be revised following processes of beliefs updating according to predictive coding principles, giving rise to posterior beliefs. Such processes involve continuous comparisons between bottom-up signals and top-down predictions, which are weighted based on their own uncertainty (or formally its inverse, precision^84^) or their reliability^85,86^. Crucially, studies in computational psychiatry suggest that biases in how people estimate *uncertainty* could explain psychopathological symptoms^87–89^. In the case of AN, we observe a strong intolerance to uncertainty^90–92^, a dimensional construct characterised by negative beliefs and reactions in response to uncertain situations or events. This trait represents a risk and maintaining factor for the development of EDs (see review^93^) and it has been strongly linked to anxiety symptoms^94^. Thus, we can appreciate how such mechanisms at the heart of the predictive coding system can be applied at multiple levels to provide a mechanistic understanding of some of the core manifestations of the disorder. In particular, embodied accounts of active inference have highlighted that we hold predictions not just about the world, but also about bodily signals^95^. If we apply such principles to the present study, we can see that when people with High-SED and AN perceive the Low-Frequency sound, which is compatible with having a heavier body, they also experience their body as heavier. We hypothesize that this may be because this perceptual experience is in line with their own attitudinal and perceptual expectations about their own body being heavy. Following this hypothesis, in this scenario the prediction error would be minimized and the experience of *“I am heavy”* meets their expectations. We also hypothesize that, in contrast, the High-Frequency condition that is usually associated with the experience of being lighter, may give rise to larger predictions errors and that these are interpreted as having lower precision (or higher uncertainty). Following this hypothesis, in this scenario such larger prediction errors may in turn be solved by an overreliance on the prior (*“I am heavy”*) rather than via processes of belief updating (which will result in the perceptual experience *“I am light”*) . Nevertheless, this only remains a temptative interpretation of our findings and a potential basis for future ad-hoc experiments since we have not specifically measured uncertainty or precision of the sensory experience in this study.

This study is particularly timely in contributing to the ongoing debate concerning the presence of body image concerns and consequent distortions of body-representation in people with EDs^2,3,7,10^. Here, we showed that eating and body image concerns, as measured by means of the Eating Disorders Examination Questionnaires, might modulate the online process of updating body-representation. Indeed, both the High-SED and AN groups, characterised by high levels of eating and body image concerns, responded to the manipulations related to the auditory driven body-weight illusion in a similar fashion, showing an inverted pattern of results to that observed for the Medium- and Low-SED groups. Our baseline measure showed deviations between the represented and the actual body weight both in the High-SED and in the Medium-SED groups^26^, but these deviations were in the direction of underestimation of body-size. These deviations were not observed in the Low-SED group, and importantly, nor in the AN group, when considering the group median. It should be noted that our clinical sample included AN inpatients who were undergoing long-term treatment in a specialised EDs centre, targeting—among others—visual body related distortions, which might have influenced the body distortions measured in the present study. Indeed, we did not collect up-to-date EDE-Q data for the AN group and therefore it is not possible to know how and to what extent the current treatment had affected their ED symptomatology. Nevertheless, individual differences in baseline body distortions contributed significantly to the observed effects, in the sense that, for instance, AN people who perceived their body as heavier at baseline, were more susceptible to the sound manipulation, specifically in the High Frequency condition. A similar pattern of results was found for the gait measure, where people with AN who perceived their body as heavier at baseline, also showed less foot acceleration in the High Frequency condition, a behaviour consistent with having a heavier body.

To our knowledge, this is the first study to investigate auditory-driven manipulations of body perception in relation to subclinical and clinical ED symptomatology and consequent behavioural and emotional changes. The intensity and persistence of body image distortions is a negative prognostic factor for long-term outcome in AN^13^, and people who have recovered from AN tend to show distorted exteroceptive and interoceptive bodily experiences suggesting that these might be more trait rather than state characteristics^96,97^. Thus, a better understanding of the malleability of body-representations in people with heightened ED symptomatology in response to sensory bodily inputs is important to improve their quality of life. Current treatments for perceptual and attitudinal body image distortions are strongly focussed on the visual experience of the body, but body image distortions are a multisensory phenomenon, as confirmed by our results, and thus treatments should take into account this multifaceted nature of body image distortions. Our study is relevant because it advances our understanding of the phenomenon in this direction. By suggesting the potential use of auditory-driven paradigms, we may open the possibilities to develop novel tools to be used in combination or alternatively to current methods for an early identification or treatment of body image distortions by targeting attitudinal and perceptual biases in response to auditory manipulations of body representation. This novel approach could be particularly useful in situations when visual exposure to the body is not possible (e.g., in blind participants) or is not feasible (e.g. high anxiety associated with direct view of the body). The use of auditory feedback in this case, offers a few interesting advantages, as apart from removing the need for direct visual contact with the body, it can provide a continuous flow of information, as audition never “turns off” in the same way that vision is blocked when closing our eyes, and it does not interfere directly with movement. By considering the unfolding opportunities for altering people’s body-representations through technologies integrating bodily auditory-feedback (see for instance^98,66^ for visual-feedback approaches), the present pilot study aimed to establish the potential value of using auditory-feedback for enhancing body-perceptions and related emotional state in relation to body image concerns (see^50,51^, testing the potential of auditory driven body-weight illusions in populations with chronic pain and stroke).

As far as we know, this is also the first study to have used the scores from the Eating Disorders Examination Questionnaire to pre-select the three groups (High, Medium, Low) of healthy participants based on their ED symptomatology. Thus, we would like to acknowledge as a limitation the lack of previous studies supporting such recruitment strategies to select participants (see^99^ for a similar approach using the EDI questionnaire). In our study, being a pilot study and hence exploratory in its nature, we used the EDE-Q score as covariate in Experiment 1 to explore the patterns of variance within each group in relation to the effects investigated. While this could be considered valid for exploratory studies, it could be considered a potential confound in the analyses, as the three groups of participants were formed based on the EDE-Q score^100^. Based on our results it is reasonable to speculate that the variation in EDE-Q between participants should be considered in relation to the effects. Any future larger study should be designed to recruit a larger set of participants and ensure that their EDE-Q score follows a continuum, in order to be able to perform regressions between the EDE-Q score and the variables of interest. We hope that this pilot study will pave the way for future investigation in the diverse range of subclinical or sub-threshold EDs, with a particular focus on different age ranges (including girls and adolescents) and across different types of EDs. We also would like to acknowledge as a limitation the modest sample size of Experiment 2; nevertheless, we were still able to find meaningful findings, and this speaks in favour of the strong effect of our experimental manipulation. Moreover, any comparisons between Experiment 1 and 2 should be taken with caution, given the heterogeneity of the population samples, as they were from different countries and environments (healthy sample tested in the lab vs. clinical sample tested in the ED centre), and the language in which the questionnaires were worded was also different (English vs. Spanish). Future studies should investigate the effect of the employed auditory driven body-weight illusion in people who have recovered from AN to obtain a broader picture of body-representation and body malleability throughout the development of the disorder (see^97^ for a similar approach), as well as investigate differences according to people’s country of origin and experimental environment.

## Conclusions

This pilot study contributed to provide a better understanding of body image distortions in people with enhanced SED and people with AN. Recent studies suggest that people with AN may be particularly captured by visual images of bodies, at the expense of internal feelings and signals. Here, we provide the first evidence for an abnormal multisensory integration of auditory (i.e., footsteps sounds signalling body size/weight) and visual (i.e., avatar’s size/weight in the body visualization tool) signals in people with subclinical SED, as well as people with AN. Re-balancing the relationship between exteroceptive bodily signals (e.g., vision and audition), can help the therapeutic path by improving the body image concerns and distortions in people with AN as well as prevent the development of such disorders in people with High-SED, perhaps targeting ages when they might be more susceptible to physical as well as emotional changes.

## Supplementary Information

Supplementary Information 1.

Supplementary Information 2.

Supplementary Information 3.

## Data Availability

All data generated or analyzed during the experiments is available in the Supplementary Material.
