# Supplementary material for: Body weight distortions in an auditory-driven body illusion in subclinical and clinical eating disorders
Source: Sci Rep. 2022 Nov 21;12:20031. doi: 10.1038/s41598-022-24452-7 (PMC9681758; doi:10.1038/s41598-022-24452-7)
Supplement: Supplementary file 1 — Supplementary Information 1. [file 41598_2022_24452_MOESM1_ESM.docx]

**ONLINE SUPPLEMENTARY MATERIAL**

This document contains supplementary methods, analyses, tables and figures accompanying the paper entitled:

**Body weight distortions in an auditory-driven body illusion in subclinical and clinical eating disorders**

Authors: Ana Tajadura-Jiménez, Laura Crucianelli, Rebecca Zheng, Chloe Cheng, Judith

Ley-Flores, Mercedes Borda-Mas, Nadia Bianchi-Berthouze and Aikaterini Fotopoulou

**SUPPLEMENTARY METHODS**

**EXPERIMENT 1: Effects of altered sound feedback in healthy individuals with Low, Medium and High symptomatology of ED**

***Participant inclusion, pre-screening and group assignment criteria***

Inclusion criteria were: age 18-35 years; normal (or corrected to normal) hearing and sight; willing to mostly stand upright for about 30 minutes, and walk for 7 periods of 1 minute each (with breaks in between); no physical condition affecting physical activity (heart disease, physical injuries, pain); no neurological/psychiatric disorder. Participants conforming these inclusion criteria were pre-screened for disordered eating and body image concerns by means of the Eating Disorders Examination Questionnaire (EDE-Q 6.0; Fairburn & Beglin, 1994, 2008). Participants that, according to their EDE-Q score, fell into any of the groups we had defined for the study (Low-, Medium or High- Symptomatology of Eating Disorders (SED), see below for selection and group assignment criteria) were invited to take part in the study, and the rest of participants were not recruited for the study. The EDE-Q has good consistency and reliability (global score α = .90; Peterson et al., 2007), and consists of 28 items rated on a 7-point Likert scale (ranging from no days/not at all to every day/markedly) and six items asking about frequency of behaviour. The questionnaire items constitute four subscales (dietary restraint, eating concern, weight concern, shape concern), and they are averaged to obtain a single global measure.

We based our criteria to select and assign participants to the three groups on previous studies which have tested the validity of the EDE-Q scores in non-clinical samples. Specifically, we set the cut-off indicative of “High-SED” at EDE-Q global score of 2.80, which is considered as the optimal cut-off score for screening EDs in primary care, according to a US-based study on 257 young adult women which aimed to test the predictive validity of the EDE-Q as a screening tool (Mond et al., 2008). A global EDE-Q score ≥2.8 has been shown to provide an optimal trade-off between sensitivity and specificity when it is used for screening in primary care (Mond et al., 2004). The mean global score for the EDE-Q has been identified as 1.52 in a study with 5255 Australia-based young adult women (Mond, Hay, Rodgers & Owen, 2006) and as 1.74 in a study with 723 US-based young adult women (Luce, Crowther & Pole, 2008). Therefore, here we choose 1.63 as the average global score (average of 1.52 and 1.74 from the above studies) to identify the “Medium-SED” group; this group was recruited by selecting the target global score +/- 0.75. As for the “Low-SED” cut off, we selected the EDE-Q range so that the three groups were balanced in their distribution. Thus, we selected 0.46 as the cut-off for the Low-SED group, which is equidistant to 1.63 as 2.80. Following these criteria, we recruited three groups of participants with Low-SED (N=21; EDE-Q score: Mean=0.27, SD=0.11, range: 0.14-0.44), Medium-SED (N=19; EDE-Q score: Mean=1.5, SD=0.36, range: 0.9-2.15) and High-SED (N=18; EDE-Q score: Mean=3.76, SD=0.59, range: 2.99-5.19). There were no significant differences in age between groups.

**EXPERIMENT 2: Effects of altered sound feedback in clinical population with AN**

***Participant characteristics***

Fifteen women (mean age ± s.d.: 26.53 ± 9.8 years; age range: 18–49 years), naïve to the study aim, were recruited from the inpatients unit at the ABB-Center (“Centro de Prevención y Tratamiento de Anorexia y Bulimia”) in Sevilla, Spain. All patients met the Diagnostic and Statistical Manual of Mental Disorders, 5th Edition (DSM-V; American Psychiatric Association, 2013) criteria for AN, as assessed using the Structured Clinical Interview for DSM-IV Axis I Disorders (SCID). Exclusion criteria included having bulimia nervosa or any other specified and unspecified eating disorders, a BMI above 18.5, being a male and any substance abuse (i.e., drug and alcohol). Our sample had an average BMI of 17.17 (± 1.03, range 14.39-18.32). The average lowest weight reached (since they have been the same height as the current one) was 38.51 kg (± 5.8 kg) and the highest was 59.24 kg (± 10.9 kg). The average length of illness was 4 years (± 2.15 years). Comorbidities included: obsessive compulsive disorder (four participants), anxiety (three participants) and depression (three participants). Six participants were on regular antidepressant medications (fluoxetine, citalopram, sertraline), but they were not at the beginning of the treatment. One participant had a history of self-harm.

**SUPPLEMENTARY ANALYSES**

**EXPERIMENT 1: Effects of altered sound feedback in healthy individuals with Low, Medium and High symptomatology of ED**

***Baseline measures: Body weight distortion, emotional state and body feelings***

We collected a baseline measure of body visualization distortion to understand whether there were any baseline differences in how our target group (i.e., High-SED) visualized their body size in comparison with the control groups. Table S1 below shows the mean (SD) actual weight, estimated weight based on visualization and baseline body image distortion (i.e., weight differential) for each SED group. Our results show that all groups underestimated their body weight in the Body Visualization task; however, the weight differential is larger for the High-SED compared to the Low-SED group (Table S1). Nevertheless, the difference in the weight differential (our index of *body image distortion*) at baseline between groups did not reach significance (*F*(2,55)=1.56, *p*=0.22). Given the large SD, we considered it important to include this weight differential as a covariate in subsequent analysis to control for its potential effect on the way participants experienced the body-weight illusion.

**Table S1.** Mean actual weight (SD), estimated weight (SD) and weight differential (SD) of participants according to their SED group. *marks significant differences between SED groups.

| **Variable** | **Low-SED (N=21)** | **Medium-SED (N=19)** | **High-SED (N=18)** |
| --- | --- | --- | --- |
| **Actual Weight (Kg)** | 53.14 (6.99) | 57.32 (7.16) | 65.11 (14.32) |
| **Estimated Weight (Kg)** | 53.05 (14.53) | 50.74 (13.38) | 57.14 (13.63) |
| **Weight Differential (Kg)** | 0.095 (17.98) | 6.58 (9.16) | 7.97 (16.20) |

  In terms of questionnaires on body feelings and emotional state at baseline, we predicted baseline differences between the High-SED group as compared to both Low- and Medium-SED groups, in line with the hypothesis of increased negativity and altered body sensations in the High-SED group. There were significant differences between SED groups for felt **Weight** (*F*(2,55)=17.77, *p*<0.001), **Feet localization** (*F*(2,55)=8.60, *p*<0.001) and **Happiness** (*F*(2,55)=4.50, *p*=0.015). There was also a trend to significance regarding **Agency** over their usual footstep sounds (*F*(2,55)=3.10, *p*=0.053 (see Table S2 below for median (range) scores of the different items of the questionnaires).

The High-SED group reported a significantly higher felt weight at baseline than the Low-SED group (*t*(55)=5.87, *p<*0.001) and there was a trend towards a significant difference in felt body weight between the High-SED group and the Medium-SED group (*t*(55)=2.22, *p*=0.07); the difference between the Low- and Medium-SED groups was also significant (*t*(55)=3.65, *p*=0.002). The High-SED participants reported feeling overall less agency over their produced walking sounds than the Medium-SED group (*t*(55)=2.43, *p*=0.047). The High-SED group felt significantly less happy than the Low-SED group (*t*(55)=2.81, *p*=0.018) and tended to feel less happy than the Medium-SED group (*t*(55)=2.37, *p=*0.055)*.* Finally, there were differences between the Low-SED and Medium-SED groups in terms of felt ability to localize their feet. The latter felt less able to localize their feet (*t*(55)=4.15, *p*<0.001) (Table S2).

Our findings, showing that the High-SED group felt less happy and heavier at baseline than the Low-SED group, aligns with the hypothesis that negative emotions towards their body are already present before the experiment. To further investigate this hypothesis, we conducted Spearman correlation analyses for the full group of participants with the variable emotional valence (i.e., felt happiness) at baseline and the global EDE-Q score, as well as the scores for the different EDE-Q scales. We found that the EDE-Q scores for the scales shape concern and weight concern, as well as for the global score, indeed correlated negatively with the valence/happiness score at baseline (EDE-Q global score: Spearman rho=.387, p=.003; shape concern: Spearman rho=.439, p=.011; weight concern: Spearman rho=.504 , p=.003), so the more concerned participants were with their shape and weight, the less happy they were. These results are in line with the hypothesis of increased negativity towards one’s body in the participants scoring high in the EDE-Q.

**Table S2.** Median (Range) for questionnaire data (7-level Likert items - scores ranging from 1-7 - except for 9-level valence, arousal and dominance scales - scores ranging from 0-8) for the three SED groups. * marks significant median differences between groups.

| **Measures** | **Low-SED (N=21)** | **Medium-SED (N=19)** | **High-SED (N=18)** |
| --- | --- | --- | --- |
| Speed | 4 (1-6) | 4(3-5) | 4(2-6) |
| **Weight*** | **4 (2-5)** | **5(3-6)** | **5.5(4-7)** |
| Strength | 5 (3-6) | 5(2-6) | 5(3-6) |
| Straightness | 4 (4-6) | 4(2-6) | 4(3-6) |
| Agency | 6 (1-7) | 5(2-7) | 6(4-7) |
| Vividness | 3 (1-5) | 4(1-6) | 3(1-5) |
| Surprise | 2 (1-5) | 4(1-6) | 3(1-6) |
| **Feet localization*** | **6 (2-7)** | **5(2-6)** | **5(1-7)** |
| **Valence (Happiness)*** | **6 (3-8)** | **5(4-7)** | **4(2-8)** |
| Arousal | 4 (0-5) | 4(0-6) | 4(0-6) |
| Dominance | 4 (3-6) | 4(3-7) | 4(1-6) |

***Effect of Sound Condition on Body Feelings and Emotional State***

We predicted that in the “High Frequency” condition, participants will report feeling their body lighter (and probably also quicker, stronger, with a more upright posture), and changes in their emotional state (feeling happier, excited, dominant). Our hypothesis was that such effects would be enhanced in the population with heightened symptomatology of EDs (SED) as compared to both Low- and Medium-SED. In order to test this hypothesis, as specified in the “Data Analysis” section in the main manuscript for questionnaire data, we conducted non-parametric ANCOVAs on aligned rank transform (ART) data, with sound condition (‘Control’, ‘High frequency’ and ‘Low frequency’) as within-subject factor, SED group as a between-subjects factor and with EDE-Q global score and body image distortion as covariates, using the R package ‘npsm’ (Kloke and McKean, 2014). In case of significant main effects of sound condition or of SED group, these were followed by t-tests on aligned rank transform data, with the p-value adjusted with the recommended Tukey method for comparing a family of 3 estimates (Wobbrock, Findlater, Gergle, & Higgins, 2011). Table S3 shows the Median (Range) for all items for the three groups of participants and the three sound conditions, for visualization purposes. Table S4 shows the Median (Range) for all items for the whole group of participants and the three sound conditions.

**Table S3.** Median (Range) for questionnaire data (7-level Likert items except for 9-level valence, arousal and dominance scales) for the three SED groups and the three sound conditions.

| Measures | Low SED participants | | | Medium SED participants | | | High SED participants | | |
| --- | --- | --- | --- | --- | --- | --- | --- | --- | --- |
|  | Control | Low freq | High freq | Control | Low freq | High freq | Control | Low freq | High freq |
| Speed | 5(2-6) | 4(1-6) | 5(2-7) | 5(3-7) | 4(1-6) | 5(2-6) | 4.5(2-7) | 4(1-6) | 5.5(2-6) |
| Weight | 4(2-6) | 5(2-6) | 3(1-6) | 4(3-5) | 5(3-6) | 4(1-6) | 4(2-6) | 5(3-7) | 3.5(2-6) |
| Strength | 4(3-6) | 4(3-6) | 4(3-6) | 4(3-6) | 5(3-6) | 5(1-6) | 4(2-6) | 4(1-6) | 4(2-6) |
| Straightness | 4(2-6) | 4(2-6) | 4(3-7) | 4(3-6) | 4(2-6) | 4(3-6) | 4(2-6) | 4(2-6) | 4(2-6) |
| Agency | 6(4-7) | 6(2-7) | 6(3-7) | 6(3-7) | 6(2-7) | 5(2-7) | 7(2-7) | 6(3-7) | 6(2-7) |
| Vividness | 4(1-6) | 3(1-6) | 3(1-6) | 3(1-5) | 3(1-6) | 3(1-7) | 4(2-6) | 4(1-5) | 3(1-7) |
| Surprise | 4(1-7) | 3(1-7) | 4(1-6) | 4(1-5) | 4(1-6) | 4(1-7) | 3(2-6) | 4(1-6) | 4(3-6) |
| Feet localization | 6(3-7) | 6(2-7) | 6(3-7) | 5(2-7) | 5(3-7) | 5(3-7) | 6(1-7) | 5(1-7) | 5(1-7) |
| Valence | 5(2-8) | 5(2-8) | 5(3-8) | 5(3-7) | 4(3-7) | 5(3-7) | 4(2-8) | 3.5(2-8) | 4(2-8) |
| Arousal | 3(0-6) | 3(0-6) | 3(0-6) | 4(0-7) | 4(0-6) | 5(0-6) | 3.5(0-6) | 4(0-6) | 4(0-6) |
| Dominance | 4(1-6) | 4(2-6) | 4(2-7) | 4(2-6) | 4(3-6) | 4(3-6) | 4(2-8) | 4(2-8) | 4(3-8) |

**Table S4.** Median (Range) for questionnaire data (7-level Likert items except for 9-level valence, arousal and dominance scales) for the whole group of participants and the three sound conditions. * marks significant median differences.

| **Measures** | **All participants** | | |
| --- | --- | --- | --- |
|  | **Control** | **Low freq.** | **High freq.** |
| **Speed*** | **5(2-7)** | **4(1-6)** | **5(2-7)** |
| **Weight*** | **4(2-6)** | **5(2-7)** | **3(1-6)** |
| Strength | 4(2-6) | 4(1-6) | 4(1-6) |
| Straightness | 4(2-6) | 4(2-6) | 4(2-7) |
| **Agency*** | **6(2-7)** | **6(2-7)** | **6(2-7)** |
| Vividness | 4(1-6) | 3(1-6) | 3(1-7) |
| Surprise | 4(1-7) | 4(1-7) | 4(1-7) |
| Feet localization | 6(1-7) | 5(1-7) | 5(1-7) |
| Valence (Happiness) | 4(2-8) | 4(2-8) | 5(2-8) |
| **Arousal*** | **4(1-7)** | **4(1-6)** | **4(1-6)** |
| Dominance | 4(1-8) | 4(2-8) | 4(2-8) |

The analyses revealed significant effects for Speed, Weight, Agency and Arousal. For Speed, there was a significant main effect of sound condition (*F*(2,110)=5.08, *p*=0.007), but no main effect of SED group nor significant interaction between factors, and no significant interaction of the main factors with the covariates (all F between 0.06 and 0.9; all p > 0.41) . As shown in Table S4 and Figure S1, participants felt overall quicker in the Control than in the Low Frequency condition (t(110)=2.86, p=0.014) and in the High Frequency than in the Low Frequency condition (t(110)=4.08, p<0.001), with no significant difference between the High Frequency and the Control conditions (p=0.44).

For Weight, there was a significant main effect of sound condition (*F*(2,110)=10.10, *p*<0.001), but no main effect of SED group nor significant interaction between factors, and no significant interaction of the main factors with the covariates (all F between 0.3 and 1.56; all p > 0.21). As shown in Table S4 and Figure S1, participants felt overall lighter in the Control than in the Low Frequency condition (t(110)=4.07, *p*<0.001) and in the High Frequency than in the Low Frequency condition (t(110)=5.59, *p*<0.001), with no significant difference between the High Frequency and the Control conditions (p=0.28).

For Agency, there was a significant main effect of sound condition (*F*(2,110)=6.98, *p*=0.001), and of SED group (F(2,55)=6.98, p=0.001), as well as a significant interaction between sound condition and SED group (F(4,110)=3.49, p=0.009). There was no significant interaction between the main factors and the covariates (F < 0.1; p = 0.99). Post-hoc paired comparisons to follow up the significant main effects and interaction, were not significant (all *p*s > 0.15). Note that while Table S4 shows the same median and range for the three sound conditions, these values were independent of the participant SED group and the within-group variability taken into account when adding the covariates. The mean (+/- SE) values of agency differed slightly across the three sound conditions - Control: 5.76 (.17), Low: 5.64 (.19), High: 5.45 (.19). In Figure S1 the significant interaction between sound condition and SED group for the agency score can be observed.

As for emotional state, for Arousal, there was a significant main effect of SED group (F(2,55)=10.69, p<0.001), but no main effect of sound condition nor significant interaction between factors, and no significant interaction of the main factors with the covariates (all F between 0.004 and 1.17; all p > 0.31). As shown in Table S4 and Figure S1, High and Medium SED participants felt overall more aroused than Low SED participants, though post-hoc paired comparisons between groups were not significant (all *p*s > 0.1).

For Valence, Dominance, Strength, Straightness, Vividness, Surprise and Feet localization, ANCOVA tests were not significant (all p > 0.13). The observed effects are in line with those observed in previous studies (Tajadura-Jiménez et al., 2015, 2019).

**
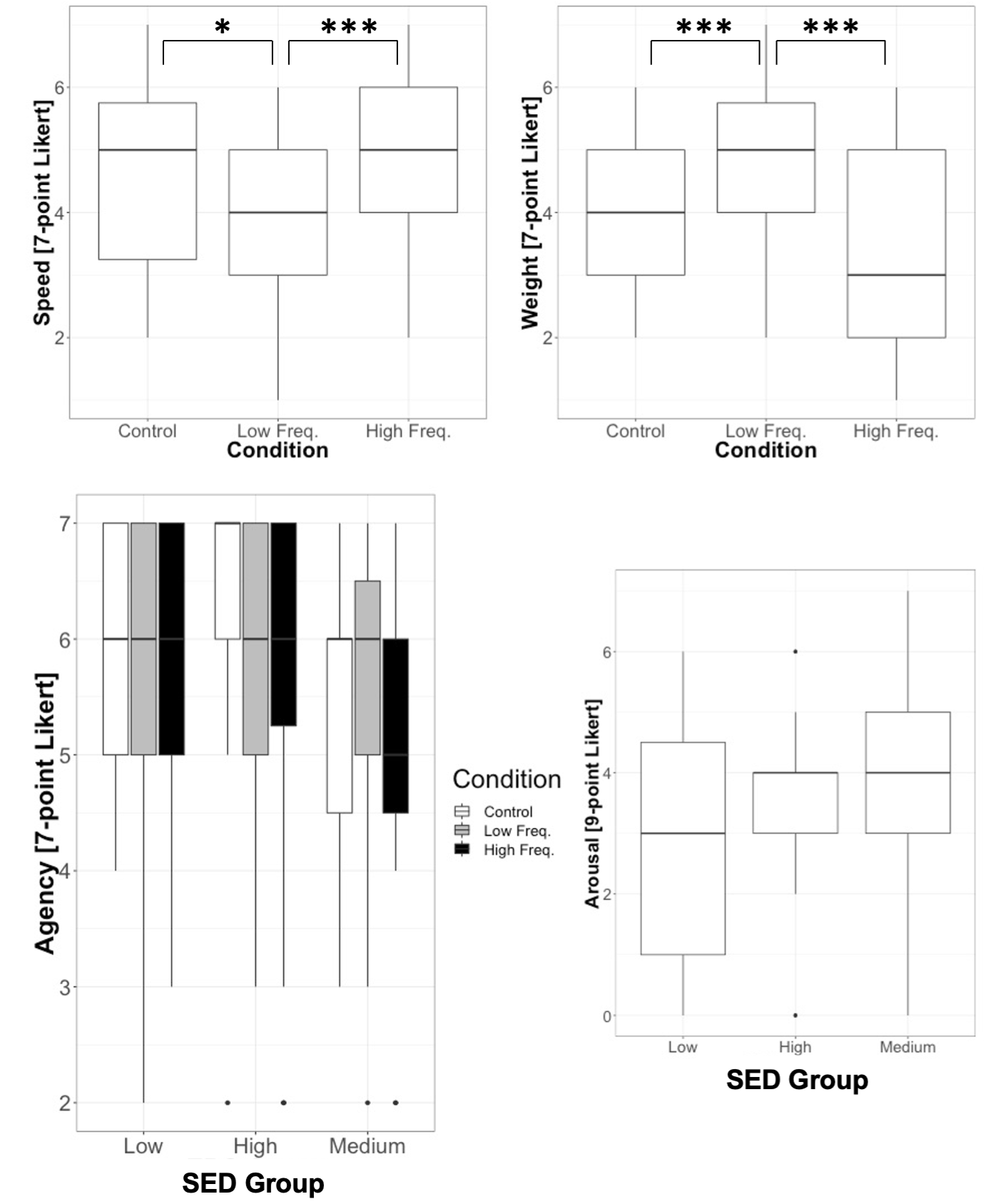
**

**Figure S1.** Median (±Range) for questionnaire items for which there were significant effects of Sound (HF-High Frequency, LF-Low Frequency, C-Control) or of SED Group (Low-, Medium-, High-SED).

***Effect of Sound Condition on Gait Patterns***

We predicted that in the “High Frequency” condition participants would adopt a behavior consistent with having a “lighter” body, characterized by more accelerated swings and less foot-ground contact time. Our hypothesis was that such effects would be enhanced in the population with heightened SED as compared to the Low- and Medium-SED. Due to technical issues, gait data was correctly recorded for only 33 participants (10 Low-SED, 10 High-SED, 13 Medium-SED). We tested our hypotheses with these data. As specified in the “Data Analysis” section, Z-scores for each gait variable were submitted to an ANCOVA with the within-subject factor sound condition, with SED group as a between-subjects group and with covariates the EDE-Q global score and body image distortion. We observed significant effects on the stride time data (analyses reported in the main manuscript) and in acceleration.

For acceleration, the main effect of sound condition (F(2,56)=3.03, p=0.056), and the interaction of sound condition with SED group (F(4,56)=2.07, p=0.093), show trends towards significance. The effects were in the same direction that those reported for the stride time data. As it can be seen in Figure S2, there were opposite patterns of the sound condition effect for the High-SED participants and the participants from the other groups. Contrary to our hypothesis, High-SED participants displayed the lowest acceleration in High- vs. Low-Frequency and Control, consistent with the stereotypical gait observed in heavier bodies. On the other hand, Low-SED participants showed the highest acceleration in High- vs. Low-Frequency, in keeping with previous studies with healthy participants (Tajadura-Jiménez et al., 2015).


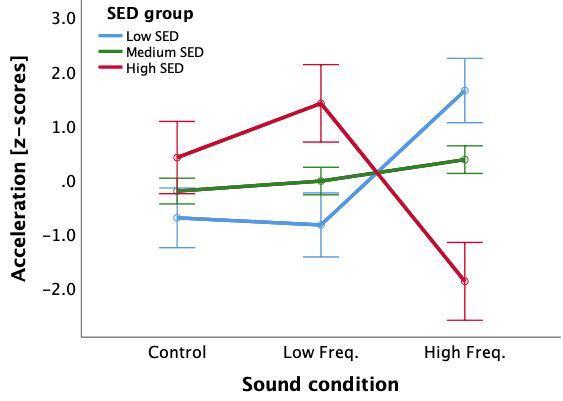


**Figure S2.** Mean(±SE) foot lift acceleration (z-scores) for all sound conditions and for each SED group, with EDE-Q global score and baseline body image distortion (i.e., weight differential) as covariates.

***Analyses without EDE-Q score as covariate***

In the analyses reported in the main manuscript and above, the EDE-Q score was included as a covariate. Note that while the different cut-offs were set to define the three SED groups according to their EDE-Q global score, the reason why we included the participant’s EDE-Q score as a covariate in the analyses was to acknowledge the importance of considering the variability between participants within each group, as the range of EDE-Q scores within each group was large.

As in the main analyses, Body Visualization and gait data were analysed with repeated measures analyses of covariance (ANCOVA), with sound condition (‘Control’, ‘High frequency’ and ‘Low frequency’) as within-subject factor, SED group as a between-subjects factor and body image distortion as covariate. The ANCOVA on the LOG-transformed Body-Visualization data showed a non-significant interaction between sound condition and SED group (F(4,108)=1.93, p=0.112, eta2=0.067). The ANCOVAs on the stride time and the acceleration data showed no significant main effects or interactions between factors (all ps > 0.38).

For questionnaire data, for the experimental conditions, we conducted non-parametric ANCOVAs on ART data using the R package ‘npsm’^72^, with sound condition (‘Control’, ‘High frequency’ and ‘Low frequency’) as within-subject factor, SED group as a between-subjects factor and body image distortion as covariate. The analyses revealed significant effects for Speed, Weight, Agency, and Arousal, which resemble those observed for the main analyses which included the EDE-Q score as covariate. In addition, the analyses revealed a significant effect for Valence. For Dominance, Strength, Straightness, Vividness, Surprise and Feet localization, ANCOVA tests were not significant (all p > 0.12).

The results for Speed, Weight, Agency and Arousal resemble those obtained for the main analyses in which the EDE-Q score was included as a covariate, with small variations in the F- and p-values. For Speed, there was a significant main effect of sound condition (*F*(2,110)=5.09, *p*=0.007), but no main effect of SED group nor significant interaction between factors, and no significant interaction of the main factors with the covariate (all F between 0.07 and 1.71; all p > 0.19). For Weight, there was a significant main effect of sound condition (*F*(2,110)=26.17, *p*<0.001), but no main effect of SED group nor significant interaction between factors, and no significant interaction of the main factors with the covariate (all F between 0.03 and 1.21; all p > 0.29). For Agency, there was a significant main effect of sound condition (*F*(2,110)=7.01, *p*=0.001), and of SED group (F(2,55)=7.01, p=0.001), as well as a significant interaction between sound condition and SED group (F(4,110)=3.50, p=0.009). For Arousal, there was a significant main effect of SED group (F(2,55)=10.7469, p<0.001), but no main effect of sound condition nor significant interaction between factors, and no significant interaction of the main factors with the covariate (all F between 0.0014 and 1.17; all p > 0.31). The follow-up analyses for these variables have been already reported above.

As for emotional state, when removing the covariate EDE-Q global score, for Valence there was also a significant main effect of SED group (F(2,55)=10.98, p<0.001), but no main effect of sound condition nor significant interaction between factors, and no significant interaction of the main factors with the covariate (all F between 0.51 and 1.79; all p > 0.17). As shown in Table S4 and Figure S3, High SED participants felt overall happier than Low SED participants (t(55)=2.73, *p*=0.023).


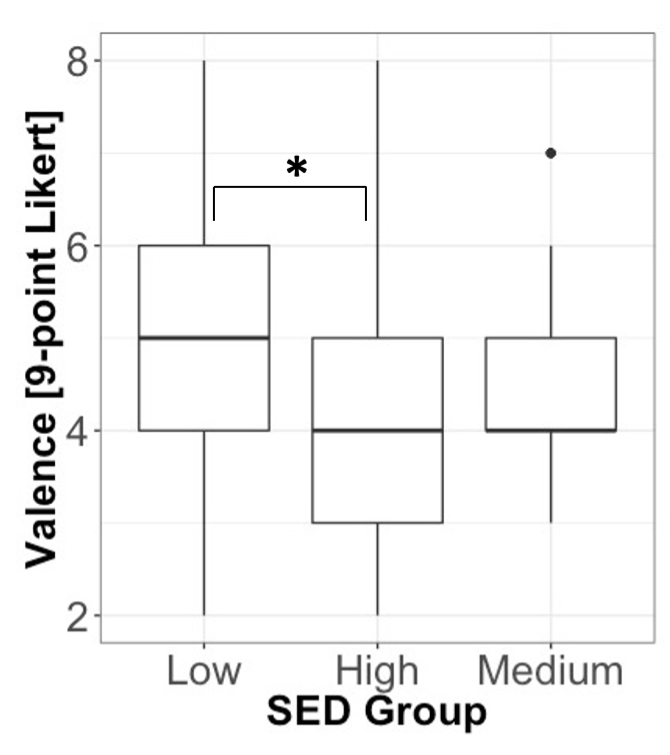


**Figure S3.** Median (±Range) for Valence for which there was a significant effect of SED Group (Low-, Medium-, High-SED) when removing the covariate EDE-Q Global score.

**EXPERIMENT 2: Effects of altered sound feedback in clinical population with AN**

***Baseline measures: Body weight distortion, emotional state and body feelings***

As in Experiment 1, we collected a baseline measure of distortions of body-representation to understand whether these distortions are present in our target group (i.e., AN). We used the body visualization tool to collect from participants their visual estimates of their body weight and calculated the differential with their actual body weight. Table S5 below shows the mean (SD) actual weight, estimated weight and weight differential (our index of body image distortion at baseline) for the AN group. The baseline body image distortion for AN participants is less than 1 Kg, while in Experiment 1 we found that for the High-SED participants the baseline body image distortion was near 8 Kg. Therefore, these participants do not show large distortions in their perceived body weight, as compared to their actual weight (as suggested in Cornelissen, Johns & Tovée, 2013). To understand if the AN group significantly differed from the High-SED group tested in Experiment 1 with regards to the body image distortion, we ran a paired t-test comparison of this variable for the two groups, which was not significant (*t*(31)=1.43, *p*=0.161).

**Table S5.** Mean actual weight (SD), estimated weight (SD) and weight differential (SD) of AN participants.

| **Variable** | **AN (N=15)** |
| --- | --- |
| **Actual Weight (Kg)** | 47.15 (7.31) |
| **Estimated Weight (Kg)** | 46.23 (16.34) |
| **Weight Differential (Kg)** | 0.92 (19.89) |

Table S6 below shows the median (range) scores of the different items of the questionnaires on body feelings and emotional state at baseline, for the AN participants. The results from the bodily feeling questionnaires, where participants had to use a 7-point Likert scale with 4 being the middle point, show that AN participants as a group felt slightly slow, heavy, weak and crouched, while they generally felt they were producing their everyday walking sounds. For the emotional feelings, measured with a 9-point Likert scale with 5 being the middle point, participants felt neutral in terms of happiness and arousal, and slightly not in control (as suggested by the dominance scale). When comparing these values with the ones for the different SED groups in Experiment 1 (see Table S2 above and full analyses comparing the four groups of participants in next section), we found that there was an overall significant effect of Group in feelings of being heavy (*F*(3,69)=9.71, *p*<0.001), strong (*F*(3,69)=4.17, *p*=0.009), up-straight (*F*(3,69)=5.15, *p*=0.002) and of being able to localize one’s feet (*F*(3,69)=6.49, *p*<0.001). Follow-up paired comparisons showed that the Low-SED group felt significantly lighter than all the other groups (High: *t*(69)=5.23, *p*<0.001; Medium: *t*(69)=3.23, *p*=0.01; AN: *t*(69)=3.36, *p*=0.007). In terms of Strength, the AN group felt significantly weaker than all the other groups (High: *t*(69)=2.76, *p*=0.036; Medium: *t*(69)=2.72, *p*=0.041; Low: *t*(69)=3.29, *p*=0.008). In terms of Straightness, the AN group felt significantly less upright than the Low-SED group (*t*(69)=3.92, *p*=0.001). In terms of Feet Localization, the Medium group felt significantly less able to localize their feet than the Low-SED group (t(69)=4.05, p<0.001) and the AN group (t(69)=3.44, p=0.005). There was an effect of Group also in terms of Happiness (F(3,69)=3.07, p=0.034) and Arousal (F(3,69)=3.25, p=0.027), with follow-up paired comparisons revealing that the High-SED felt unhappier than then Low-SED group (t(69)=2.73, p=0.049) and the AN group felt more aroused than then Low-SED group (t(69)=3.09, p=0.015). However, these comparisons should be taken with caution as the populations were from different countries and environments (healthy sample tested in the lab vs. clinical sample tested in the ED centre), and the language in which the questionnaires were worded was also different (English vs Spanish).

**Table S6.** Median (Range) for questionnaire data (7-level Likert items - scores ranging from 1-7 - except for 9-level valence, arousal and dominance scales - scores ranging from 0-8) for AN participants.

| **Measures** | **AN (N=15)** |
| --- | --- |
| Speed | 3 (1-7) |
| Weight | 5 (2-7) |
| Strength | 3 (1-6) |
| Straightness | 3 (1-6) |
| Agency | 6 (3-7) |
| Vividness | 4 (1-7) |
| Surprise | 4 (1-7) |
| Feet localization | 6 (2-7) |
| Valence (Happiness) | 4 (0-7) |
| Arousal | 4 (1-7) |
| Dominance | 3 (0-5) |

***Effect of Sound Condition on Body Feelings and Emotional State (Questionnaire Data)***

In keeping with previous studies, we predicted that in the “High Frequency” condition participants would report feeling their body lighter (probably also quicker, stronger, with a more upright posture), and changes in their emotional state (feeling happy, excited, dominant). As in the case of the High-SED group in Experiment 1, we also hypothesized that such effects would be enhanced in the population with AN, as compared to the participants in Experiment 1. In order to test the first prediction*,* as specified in the “Data Analysis” section (see main manuscript), we conducted ANCOVAs on aligned rank transform data, with sound condition as within-subject factor and with the baseline body image distortion as covariate. Table S7 shows the Median (Range) for all items and the three sound conditions.

**Table S7.** Median (Range) for questionnaire data (7-level Likert items - scores ranging from 1-7 - except for 9-level valence, arousal and dominance scales - scores ranging from 0-8) for the three sound conditions, for AN participants.

| **Measures** | **Control** | **Low freq.** | **High freq.** |
| --- | --- | --- | --- |
| Speed | 4(1-7) | 4(1-7) | 3(1-6) |
| Weight | 4(1-6) | 5(1-7) | 5(1-7) |
| Strength | 4(1-7) | 3(2-7) | 3(2-7) |
| Straightness | 4(0-7) | 4(0-7) | 4(0-7) |
| Agency | 6(1-7) | 6(3-7) | 6(3-7) |
| Vividness | 4(1-6) | 3(1-6) | 4(1-7) |
| Surprise | 4(1-7) | 4(1-7) | 5(1-7) |
| Feet localization | 5(1-7) | 5(2-7) | 4(1-7) |
| Valence (Happiness) | 4(0-8) | 4(1-7) | 5(1-7) |
| Arousal | 4(0-8) | 5(0-8) | 5(0-7) |
| Dominance | 4(1-6) | 4(0-6) | 3(0-5) |

The effect of sound condition was not significant, and the baseline body image distortion did not significantly interact with the factor sound condition for any of the questionnaire items. Nevertheless, the analyses revealed significant effects of the covariate baseline body image distortion for Strength (*F*(2,7.65)=12.55, *p*=0.001) and Agency (*F*(2,8.16)=13.57, *p*<0.001). As it can be seen in Figure S4, while there was no significant relation between sound condition and reported strength, larger baseline body image distortions (or weight differentials) predicted lower reported strength (R^2^=0.25). Furthermore, while there was no significant relation between sound condition and reported agency over the heard walking sounds, larger baseline body image distortions (or weight differentials) predicted higher reported agency (R^2^=0.23).

| 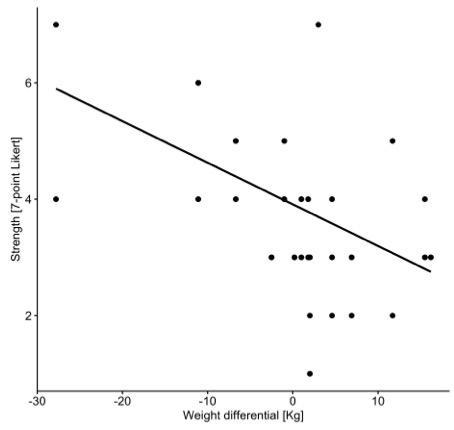 | 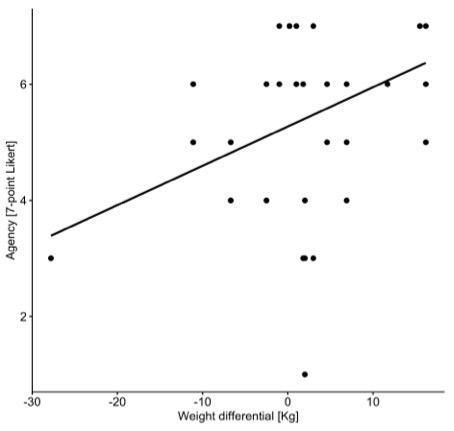 |
| --- | --- |

**Figure S4.** Correlations between weight differential (our index of body image distortions at baeseline) and reported strength (left panel) and agency (right panel).

With regards to the hypothesis of enhanced effects for the AN group, as compared to the different SED groups in Experiment 1, with the four groups of participants (Low-SED, Medium-SED, High-SED, AN), we conducted non-parametric ANCOVAs on aligned rank transform data using the R package ‘npsm’ (Kloke and McKean, 2014), with sound condition (‘Control’, ‘High frequency’ and ‘Low frequency’) as within-subject factor, group (Low-SED, Medium-SED, High-SED, AN) as a between-subjects factor, and the baseline weight differential as covariate. In case of significant main effects of sound condition or of group, these were followed by t-tests on aligned rank transform data, with the p-value adjusted with the recommended Tukey method for comparing a family of 4 estimates (Wobbrock et al., 2011).

We found that there was an overall significant effect of sound condition for Speed (*F*(2,2.49)=3.81, *p*=0.023) and Weight (*F*(2,7.86)=12.71, *p*<0.001), replicating previous results that participants felt overall quicker and lighter in the High than in the Low Frequency condition. Further, there were significant interactions between sound condition and group for Speed (*F*(6,5.19)=2.64, *p*=0.017) and Weight (*F*(6,5.53)=2.98, *p*=0.008), though follow-up comparisons were not significant. Finally, there was an overall significant effect of group for Weight (*F*(3,3.94)=4.25, *p*=0.006), Strength (*F*(3,5.25)=10.70, *p*<0.001), Agency (*F*(3,2.69)=3.45, *p*=0.017), Surprise (*F*(3,3.09)=2.95, *p*=0.033), Valence (F(3,18.18)=9.20, p<0.001) and Arousal (F(3,29.69)=25.02, p<0.001). These analyses are detailed below.

For Speed, there was a significant effect of sound condition (*F*(2,2.49)=3.81, *p*=0.023), and an interaction between sound condition and group (*F*(6,5.19)=2.64, *p*=0.017). There was no significant main effect of group nor interaction between the main factors and the covariate (all F < 1.37; all p > 0.25). Participants felt overall quicker in the High Frequency than in the Low Frequency condition (t(138)=2.82, *p*=0.015), and to some extent also quicker in the Control than in the Low Frequency condition, although that comparison did not reach significance after adjusting for multiple comparisons (*p*=0.08). With regards to the interaction between sound condition and group, this was caused by the fact that, contrary to our hypothesis, in the High Frequency condition AN participants felt slower than in the Control condition, as it can be observed in Figure S5. Nevertheless, post-hoc paired comparisons to follow up the significant interaction, did not reach significance (all *p*s > 0.47).

Similarly, for Weight, there was a significant effect of sound condition (*F*(2,7.86)=12.71, *p*<0.001), a significant effect of group (*F*(3,3.94)=4.25, *p*=0.006) and an interaction between sound condition and group (*F*(6,5.53)=2.98, *p*=0.008). There was no significant interaction between the main factors and the covariate (F < 0.1; p = 0.92). Participants felt overall lighter in the Control than in the Low Frequency condition (t(138)=3.83, p<0.001) and in the High Frequency than in the Low Frequency condition (t(138)=3.67, p=0.001), with no significant difference between the High Frequency and the Control conditions (p=0.99). With regards to the interaction between sound condition and group, this was caused by the fact that, contrary to our hypothesis, in the High Frequency condition AN participants felt heavier than in the Control condition, as it can be observed in Figure S5. Nevertheless, post-hoc paired comparisons to follow up the significant interaction and the effect of Group, did not reach significance (all *p*s > 0.32).

| 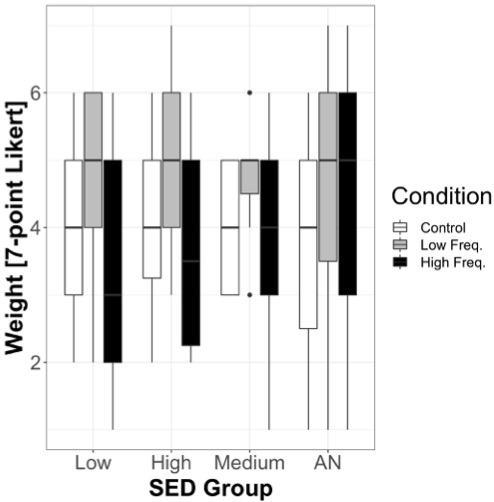 | 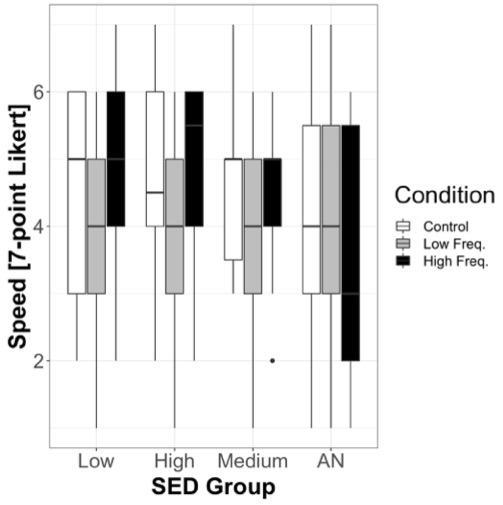 |
| --- | --- |

**Figure S5.** Median (±Range) for questionnaire items for which there were significant effects of Sound Condition (High Frequency, Low Frequency, Control) and an interaction between Sound Condition and Group (Low-SED, Medium-SED, High-SED, AN).

For Strength, there was a significant effect of group (*F*(3,5.25)=10.70, *p*<0.001). There was no effect of sound condition, nor significant interaction between the main factors or the covariate (all F < 0.1; all p > 0.99). Post-hoc paired comparisons to follow up the significant main effect of Group did not reach significance (all *p*s > 0.16).

Similarly, for Agency, there was a significant effect of group (*F*(3,2.69)=3.45, *p*=0.017). There was no effect of sound condition, nor significant interaction between the main factors or the covariate (all F < 1.72; all p > 0.17). Post-hoc paired comparisons to follow up the significant main effect of Group did not reach significance (all *p*s > 0.28).

Similarly, for Surprise, there was a significant effect of group (*F*(3,3.09)=2.95, *p*=0.033). There was no effect of sound condition, nor significant interaction between the main factors or the covariate (all F < 2.00; all p > 0.13). Post-hoc paired comparisons to follow up the significant main effect of Group did not reach significance (all *p*s > 0.49).

As for emotional state, there were significant effects for Valence and Arousal. For Valence (Happiness), there was a significant effect of group (F(3,18.18)=9.20, p<0.001). There was no effect of sound condition, nor significant interaction between the main factors or the covariate (all F < 3.59; all p > 0.059). Post-hoc paired comparisons to follow up the significant main effect of Group did not reach significance, although there were some trends to show that the High-SED participants were less happy than the Low-SED (t(69)=2.54, *p*=0.063) and the AN participants (t(69)=2.56, *p*=0.060).

Similarly, for Arousal, there was a significant effect of group (F(3,29.69)=25.02, p<0.001). There was no effect of sound condition, nor significant interaction between the main factors or the covariate (all F < 1.3; all p > 0.22). Post-hoc paired comparisons to follow up the significant main effect of Group show that the AN participants were significantly more aroused than the Low-SED (t(69)=2.99 *p*=0.020). Other group comparisons did not reach significance (all *p*s > 0.14).

**REFERENCES**

American Psychiatric Association. 2013. Anxiety disorders. In *Diagnostic and statistical manual of mental disorders* 5th ed.

Fairburn, C. G., & Beglin, S. J. (1994). Assessment of eating disorders: interview or self-report questionnaire? *The International Journal of Eating Disorders, 16*(4), 363.

Fairburn, C. G., & Beglin, S. J. (2008). *Eating disorder examination questionnaire*. In C. G. Fairburn (Ed.), Cognitive behaviour therapy and eating disorders (pp. 311–313). New York, NY, USA: Guilford Press.

Kloke, J. (2014). *Nonparametric Statistical Methods Using R / Kloke, John.* (J. McKean, Ed.) (1st edition). Chapman and Hall/CRC.

Luce, K. H., Crowther, J. H., & Pole, M. (2008). Eating Disorder Examination Questionnaire (EDE-Q): Norms for undergraduate women. *International Journal of Eating Disorders, 41(3), 273–276*. <http://doi.org/10.1002/eat.20504>

Mond, J. M., Hay, P. J., Rodgers, B., Owen, C., & Beumont, P. J. V. (2004). Validity of the Eating Disorder Examination Questionnaire (EDE-Q) in screening for eating disorders in community samples. Behaviour research and therapy, 42(5), 551-567. <https://doi.org/10.1016/s0005-7967(03)00161-x>

Mond, J. M., Hay, P. J., Rodgers, B., & Owen, C. (2006). Eating Disorder Examination Questionnaire (EDE-Q): Norms for young adult women. *Behaviour Research and Therapy, 44(1)*, 53–62. <http://doi.org/10.1016/J.BRAT.2004.12.003>

Mond, J. M., Myers, T. C., Crosby, R. D., Hay, P. J., Rodgers, B., Morgan, J. F., … Mitchell, J. E. (2008). Screening for eating disorders in primary care: EDE-Q versus SCOFF. *Behaviour Research and Therapy, 46*(5), 612–622. <http://doi.org/10.1016/J.BRAT.2008.02.003>

Tajadura-Jiménez, A., Basia, M., Deroy, O., Fairhurst, M., Marquardt, N., & Bianchi-Berthouze, N. (2015). As Light As Your Footsteps: Altering Walking Sounds to Change Perceived Body Weight, Emotional State and Gait. In *Proceedings of the 33rd Annual ACM Conference on Human Factors in Computing Systems* (pp. 2943–2952). New York, NY, USA: ACM. <https://doi.org/10.1145/2702123.2702374>

Tajadura-Jiménez, A., Newbold, J., Zhang, L., Rick, P., & Bianchi-Berthouze, N. (2019). As Light as You Aspire to Be: Changing Body Perception with Sound to Support Physical Activity. In *CHI Conference on Human Factors in Computing Systems Proceedings (CHI 2019), May 4–9, 2019, Glasgow, Scotland, UK*. ACM, New York, NY, USA.

Wobbrock, J. O., Findlater, L., Gergle, D., & Higgins, J. J. (2011). The Aligned Rank Transform for Nonparametric Factorial Analyses Using Only Anova Procedures. In *Proceedings of the SIGCHI Conference on Human Factors in Computing Systems* (pp. 143–146). New York, NY, USA: ACM. https://doi.org/10.1145/1978942.1978963
